# Paf1 and Ctr9, core components of the PAF1 complex, maintain low levels of telomeric repeat containing RNA

**DOI:** 10.1093/nar/gkx1131

**Published:** 2017-11-14

**Authors:** Joana Rodrigues, David Lydall

**Affiliations:** Institute for Cell and Molecular Biosciences, Newcastle University Medical School, Newcastle upon Tyne NE2 4HH, UK

## Abstract

The conserved PAF1 complex (Cdc73, Paf1, Ctr9, Leo1 and Rtf1, in yeast), binds RNA pol II, and affects levels of many RNAs. Although PAF1 is a complex, there is evidence that different components perform different functions. In yeast, Cdc73, Paf1 and Ctr9 maintain normal telomerase RNA (TLC1) levels and affect telomere length. Here we report a new connection between the PAF1 complex and telomere biology. We show that Paf1 and Ctr9 maintain low telomere repeat containing RNA (TERRA) levels while Cdc73, Leo1 and Rtf1 have lesser effects. Analysis of double mutants shows that Paf1 and Ctr9 can affect TERRA independently of Sir4, Rat1, and Trf4, previously identified regulators of TERRA. The data suggest that Paf1 and Ctr9 maintain low TERRA levels by affecting both transcription and degradation and that short telomeres in *cdc73Δ, paf1Δ* and *ctr9Δ* mutants do not induce TERRA. These data establish the PAF1 complex as a new regulator of TERRA, and are consistent with the model in which Paf1 and Ctr9, the core components of the PAF1 complex, affect transcript levels and cell fitness by numerous mechanisms.

## INTRODUCTION

**Figure 1.**
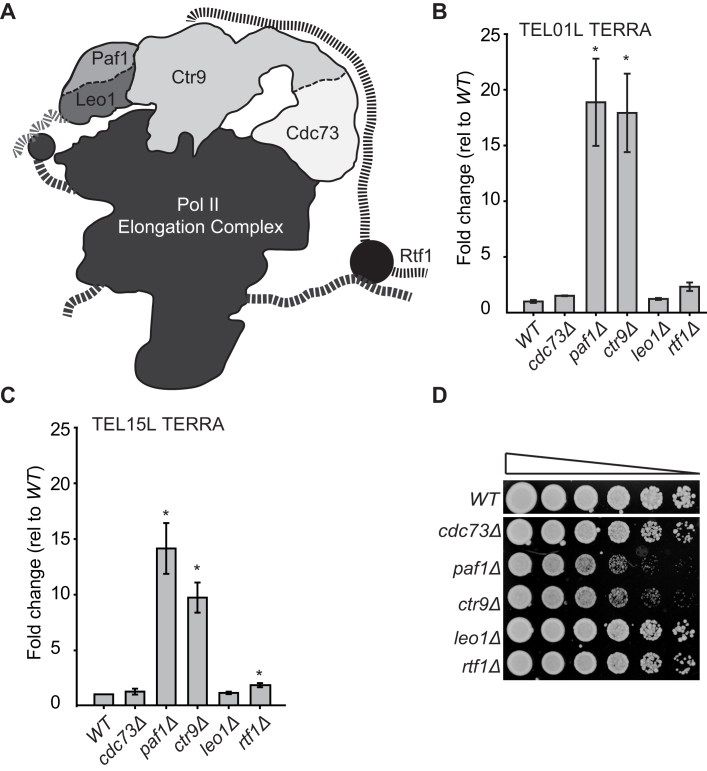
Paf1 and Ctr9 repress TERRA levels. (**A**) A cartoon based on published structural data, estimating the locations of the PAF1 complex components Cdc73, Paf1, Ctr9, Leo1 and Rtf1 on Pol II Elongation complex (12 subunit RNA pol II, TFIIS and Spt5) (3). Ctr9 acts as a bridge between Paf1-Leo1 and Cdc73 and does not strongly interact with RNA pol II, Rtf1 is flexible. Dashed lines indicate flexible regions. (**B**) TERRA from two independent strains of each genotype was measured by two-step qRT-PCR. Each value was normalized to *ACT1* mRNA and is indicated as fold change over *WT*. Error bars represent average deviation. (**C**) One-step qRT-PCR analysis of TERRA in the strains indicated, as described in B. *BUD6* mRNA was used as the internal control (40). (**D**) Serial dilutions (5-fold) of saturated overnight cultures, grown at 23°C, were spotted onto YEPD plates and incubated for 2 days at 23°C. All strains were grown on a single plate but images, where indicated, have been cut and pasted to allow better comparisons. The spot tests shown are representative of many replicate experiments. Strain numbers can be found in Table S1. Statistical analyses used the two-tailed unpaired T test (*P < 0.05).

Cdc73, Paf1, Ctr9, Leo1 and Rtf1 are components of the PAF1 complex in yeast, a transcription elongation factor (TEF), that binds RNA pol II (Figure 1A) and affects RNA levels via a number of routes, transcriptional and post-transcriptional (1–3). The PAF1 complex regulates histone modifications, promoter-proximal pausing of RNA pol II, transcript initiation, elongation, termination, polyadenylation, nuclear export and RNA stability (1,2,4–9). Perhaps not surprisingly, given its numerous roles, there is evidence that the PAF1 complex affects RNA levels both positively and negatively (3,8). Recently, the PAF1 complex was shown to function with the DNA damage response protein, Mec1 and the INO80 chromatin remodelling complex, to evict RNA pol II from chromatin, to help avoid collisions between DNA replication forks and transcription machinery (10).

Evidence from mammalian and yeast cells suggests that different subunits of the PAF1 complex perform different functions. For example, in mouse muscle cells, Rtf1 is missing from the PAF1 complex and is replaced by Ski8, an adaptor for the cytoplasmic exosome (11). In yeast, *paf1Δ* and *ctr9Δ* cells are comparatively unfit while *cdc73Δ, leo1Δ* and *rtf1Δ* cells are fit, showing that Paf1 and Ctr9 are more important for cell function than Cdc73, Leo1 and Rtf1 (12,13). Also in yeast, *cdc73Δ, paf1Δ* and *ctr9Δ* cells have short telomeres due to low levels of TLC1, the RNA component of telomerase, while *leo1Δ* and *rtf1Δ* cells have comparatively normal length telomeres and TLC1 levels (13,14). Finally, human *CDC73* and *CTR9* genes are frequently found mutated in cancer, and are the only PAF1 complex genes to be currently classified as tumour suppressors (15,16).

Interestingly, the poor fitness of *paf1Δ* and *ctr9Δ* yeast cells seems to be partially due to defective telomeres (13). This was concluded because overexpression of *TLC1* improved the fitness of *paf1Δ* and *ctr9Δ* cells and partially rescued the telomere length defects of these cells (13). However, the question remains as to why *cdc73Δ* cells, which have equally low *TLC1* levels and short telomeres as *paf1Δ* or *ctr9Δ* cells, are comparatively fit.

We hypothesized that PAF1 complex components might affect levels of other telomere-related factors that contribute to cell fitness. Telomeric repeat containing RNA (TERRA) is, like *TLC1*, a noncoding RNA that is important at telomeres, and was a candidate for PAF1 complex control. TERRA is observed in yeasts and mammalian cells but its function and regulation are not fully understood. TERRA is of biological significance because it can regulate telomerase activity, promote recombination and activate the DNA damage response (17–20). Additionally, TERRA RNA:DNA hybrids at the telomere can stimulate homologous recombination or G-quadruplex formation (17,21–23). Interestingly, TERRA levels have been found to be downregulated in telomerase-positive cancer cells, and up-regulated in ALT-dependent cancer cells (24,25).

In yeast, TERRA does not have defined promoters or terminators, and is a 5′ capped RNA pol II transcript, primarily located in the nucleus (18,19). There are 32 telomeres in budding yeast, with strong similarities but some structural differences, and the evidence is that TERRA from each is regulated somewhat differently (26,27). TERRA can be both polyadenylated and non-polyadenylated (18,28–32). The transcription of TERRA is repressed, like other telomeric transcripts, by the Sir complex, a histone deacetylase composed of Sir2, Sir3 and Sir4 (26,33). TERRA is principally degraded by Rat1, the major nuclear 5′ to 3′ RNA exonuclease (18,19,26,31,34). In addition, there is evidence that the TRAMP complex (Trf4/Air2/Mtr4p Polyadenylation complex) promotes TERRA degradation presumably in the 3′ to 5′ direction by the nuclear exosome (18,19,26,31,34).

High TERRA levels have been associated with short telomeres and vice versa (28,31,35,36). For example, induction of TERRA transcription at a telomere specifically shortened that telomere (36). Similarly, experimental shortening of a single telomere, led to increased TERRA at the same telomere (28). Since PAF1 components can affect RNA pol II transcripts by numerous mechanisms, and affect telomere length and cell fitness, it seemed plausible that TERRA levels could be affected by the PAF1 complex.

Here we show that Paf1 and Ctr9, but not Cdc73, Leo1 or Rtf1, play an important role maintaining low TERRA levels. High TERRA levels correlate with the poor fitness of *paf1Δ* and *ctr9Δ* cells. Importantly, a number of experiments show that Paf1 and Ctr9 are able to maintain low TERRA levels through mechanisms that are independent of the established mechanisms of TERRA regulation. Our evidence suggests that Paf1 and Ctr9, the core components of the PAF1 complex, affect TERRA levels transcriptionally and post-transcriptionally.

## MATERIALS AND METHODS

### Yeast strain growth and transformation

Standard procedures for yeast culture, mating and tetrad dissection were followed (37). Unless otherwise stated, all experiments were performed using *Saccharomyces cerevisiae* W303 (*RAD5*) strains. Consult S1 Table for strain genotypes. YEPD was supplemented with adenine (75 mg/l). Gene disruptions were made using one step PCR to insert kanMX or hphMX cassettes into the genome (38) and confirmed by PCR. Primers and plasmids are listed (S2 and S3 Tables).

### Yeast growth assays

A pool of colonies (>10) were grown in 2 ml liquid YEPD or SC media overnight at 23°C. 5-10-fold serial dilutions in water were spotted onto round or rectangular agar plates using a replica plating device. Plates were incubated for 2–5 days at the appropriate temperatures before being photographed.

### Analysis of telomere structure

Southern blot analysis was used to assess telomere length as previously described (39). Genomic DNA was extracted, digested with XhoI (Y’+TG and 15L probes) or HindIII (10R, and 13R probes) and then run overnight on a 1% agarose gel at 1 V/cm. Southern transfer was performed using a Biorad Vacuum Blotter according to manufacturer's indications. Probe labelling, hybridization and washing were performed according to the DIG High Prime DNA Labelling and Detection Starter Kit II (Roche) instructions. The Y’+TG probe is approximately 1 kb with ∼880 bp of Y’ and 120 bp of TG repeats and is released from pDL1574 using XhoI and BamHI. The TEL10R, TEL13R and TEL15L probes were made using primers indicated in Table S2. All probes were gel-purified before being DIG-labelled (DIG High Prime DNA Labelling and Detection Starter Kit II, Roche) and probes TEL10R and TEL15L were also digested with HaeIII prior to labelling, as suggested by the manufacturer for long DNA fragments. Loading control pictures were taken from each gel, before transfer, using SYBR Safe DNA Gel Stain to stain the total DNA (lanes were vertically compressed using Adobe Illustrator for presentation purposes).

### Quantitative RT-PCR

In most cases two-step qRT-PCR experiments were performed to measure TERRA. RNA was purified using the RNEasy Mini Kit (Qiagen) with 3 DNAse I digestions (first an ‘on-column’ digestion, then an ‘in solution’ and finally another ‘on-column’ digestion) using the RNase-Free DNase Set (Qiagen). The first, cDNA synthesis step, was performed exactly as published (26). In brief, 3 μg of RNA were reverse transcribed using a CA-rich primer (m4104), which binds all telomeric repeats, and an *ACT1*-specific reverse primer (m4103), as a loading control. The second step, qPCR of cDNA, was performed using forward and reverse primers, directed to specific telomeres, with the Platinum SYBR Green qPCR SuperMix-UDG with ROX (Invitrogen) kit in a final volume of 10 μl. RNA measurements were normalized relative to *ACT1* mRNA levels, as previously described (26,40). The comparative 2^–ΔΔCT^ method was used to plot the graphs relative to *WT* values as described before (41).

RNA for one-step qRT-PCR analysis was isolated by phenol essentially as described in (42), followed by further purification using an RNEasy Mini Kit and DNase I digestion (Invitrogen). Next, one-step qRT-PCR was carried out in the presence of forward and reverse primers, directed to specific telomeres, using the Superscript III Platinum SYBR Green One-Step qRT-PCR kit (Invitrogen). RNA measurements were normalized relative to *BUD6* RNA, amplified in a separate reaction as previously described (26,40).

For the measurement of polyadenylated TERRA, independent cDNA synthesis reactions were performed using either: (i) CA-rich and *ACT1*-specific reverse primers; or (ii) an Oligo(dT)15 primer (C1101, Promega). qPCR to detect TERRA and polyadenylated TERRA used telomere specific forward primers and the CA-rich reverse primer. In addition, the cDNA synthesis step was modified as follows: after the 90°C denaturation step, the samples were incubated 1 min at 42°C, the enzyme mix was added and the samples were further incubated at 42°C for 6.5 min (according to Oligo(dT)15 manufacturer's instructions). The extension step was performed at 55°C for 60 min.

### RNA half-life determination

An anchor-away strain background (tor1–1 fpr1::NAT RPL13A-2*FKBP12::TRP1 Rpb1-FRB-kanMX6) was used to stop RNA pol II-mediated transcription upon rapamycin addition (43). Briefly, Rpb1, the main subunit of RNA pol II, is fused to the human FKBP12-rapamycin-binding (FRB) domain of mTOR. FKPB12 is fused to Rpl13a, a yeast ribosomal protein. Upon rapamycin addition FRB and the FKBP12 domains interact and the Rpb1 fusion is moved to the cytoplasm. For RNA isolation, cell cultures were grown overnight at 30°C until early exponential phase (OD600 ∼ 0.5). A fraction of the culture was harvested (0 min time-point) and rapamycin was added (1 μg/ml final concentration). Total RNA was isolated from cells harvested at 0, 10, 20, 40 and 60 min after rapamycin addition. RNA levels were measured for each time-point and normalized for the amount of 7S RNA. The amount of RNA is plotted (log_2_) as the fold change in relation to the amount of RNA at time point 0. The slope (m) of the line that best fitted the data, ignoring the 0 minute time point, was used to calculate the RNA half-life using the equation *T*_$\frac{1}{2}$_ = 0.693/m (26).

The transcription inhibitors 1,10-phenanthroline (100 mg/ml in 50% ethanol) or thiolutin (5 mg/ml in DMSO) were used at final concentrations of 100 μg/ml or 3 μg/ml (44–46). RNA isolation and normalization was performed as described above, for the anchor-away system. The amount of RNA was plotted as the percentage of the RNA levels observed at time point 0.

### Statistical analyses

For qPCR and Southern blot measurements the mean of the two independent strains is shown and the error bars, or average deviation, represent the range of the two individual values. Statistical analysis used the two-tailed unpaired *t*-test (*P < 0.05, **P < 0.01 and ***P < 0.001).

### Chromatin immunoprecipitation (ChIP)

ChIP was performed as previously described with the minor modification that formaldehyde crosslinking was performed for 60 min (47). 3 μg of mouse anti-FLAG (Sigma F3165) or goat anti-mouse (Dako P0447) antibodies were used per sample. Immunoprecipitated DNA was quantified by qPCR using a SYBR Green qPCR SuperMIX-UDG w/ROX kit (Invitrogen).

## RESULTS

### Paf1 and Ctr9 are needed for cell fitness and low TERRA levels

Of the 32 telomeres in yeast, approximately half are X telomeres, lacking the repetitive Y’ element found on the remaining chromosome ends, and it is possible to generate specific PCR primers to these ends. To determine whether TERRA is affected in PAF1 complex mutants, we measured TERRA transcribed from left-arm X telomeres on chromosomes 1 and 15 (TEL01L and TEL15L), where TERRA had previously been measured (26,36). Strikingly, *paf1Δ* and *ctr9Δ* strongly increased TERRA, while *cdc73Δ, leo1Δ* and *rtf1Δ* had smaller effects (Figure 1B, C). We confirmed, as reported by others, that *paf1Δ* and *ctr9Δ* cells are comparatively unfit (deduced by size of colonies on agar plates), while *cdc73Δ, leo1Δ* and *rtf1Δ* are fit (Figure 1D) (12). Therefore, high TERRA levels in *paf1Δ* and *ctr9Δ* cells correlate with the poor fitness of these mutants. Indeed, high TERRA seems to correlate better with poor fitness than low *TLC1* levels because *TLC1* levels are similarly low in *cdc73Δ, paf1Δ* and *ctr9Δ* cells, but only *paf1Δ* and *ctr9Δ* are noticeably unfit (Supplementary Figure S1 and Figure 1D).

To determine if Paf1 and Ctr9 affect polyadenylated and non-polyadenylated TERRA similarly, total and polyadenylated TERRA levels in *paf1Δ* and *ctr9Δ* cells were measured using the method described in the scheme in Supplementary Figure S2A. Polyadenylated and non-polyadenylated TERRA are equally increased in *paf1Δ* and *ctr9Δ* mutants (Supplementary Figure S2B). We estimate that 7% of TERRA is polyadenylated, similar to the findings in mammalian and *Schizosaccharomyces pombe* (Supplementary Figure S2C) (30,32,48), but lower than previous estimates in budding yeast *rat1–1* mutants (31). We conclude that Paf1 and Ctr9 strongly repress polyadenylated and non polyadenylated TERRA levels and that this effect correlates with the poor fitness of *paf1Δ* and *ctr9Δ* mutants.

### Paf1 and Ctr9 regulate TERRA independently of telomere length

Short telomeres have been associated with high TERRA levels (20,28,31,36,49). However, *cdc73Δ, paf1Δ* and *ctr9Δ* cells have similarly short telomeres, and low levels of *TLC1*, but *cdc73Δ* cells have normal levels of TERRA in comparison with *paf1Δ* and *ctr9Δ* cells (Supplementary Figure S3B and Figure 1B and C). To better understand the correlation between telomere length and TERRA levels in *cdc73Δ, paf1Δ* and *ctr9Δ* mutants, the lengths of specific telomeres and TERRA levels from those same telomeres were examined from the same cultures. As controls, *sir4Δ* strains, previously reported to contain increased TERRA levels and *yku70Δ* and *mre11Δ* strains with short telomeres were examined (26,28). At the same time, we tested the effects of deleting *CDC73* in *paf1Δ* and *ctr9Δ* cells, since Cdc73 is required for the physical interaction of the PAF1 complex with RNA pol II, and in *Drosophila* Cdc73 aids transcription of Wnt target genes (2,25,31,50). This led us to hypothesise that Cdc73 might be important for TERRA transcription.

**Figure 2.**
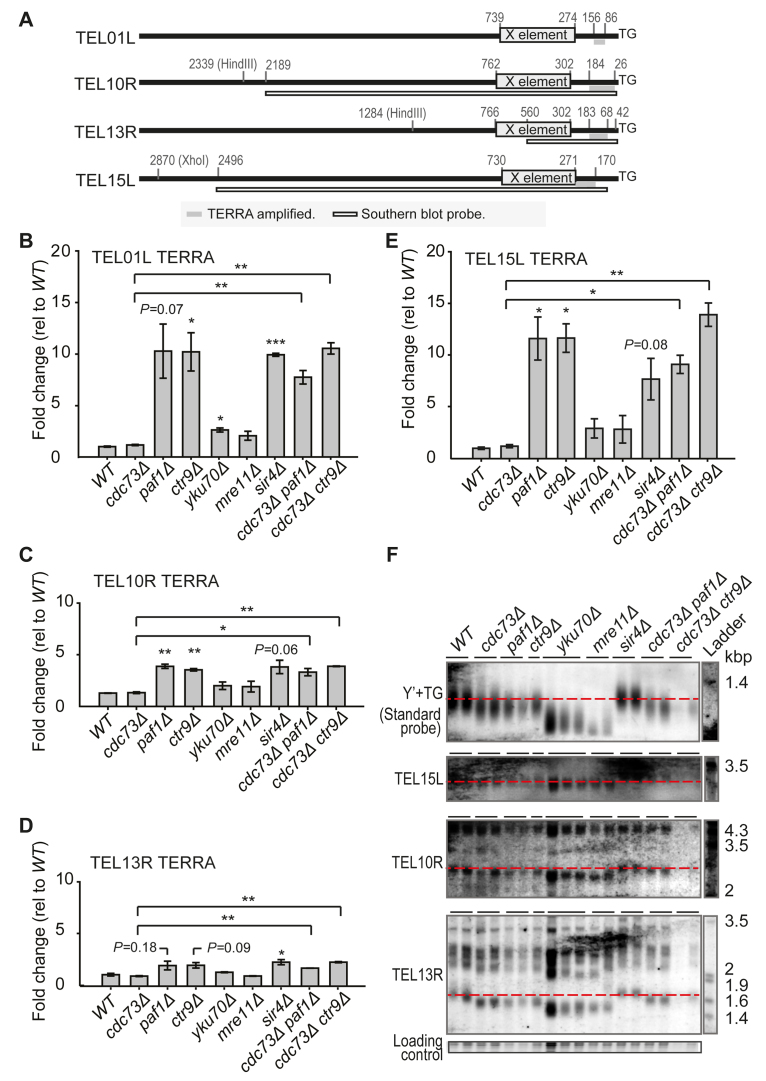
Lack of correlation between telomere length and TERRA levels in cells with stable telomeres. Two independent, exponentially-dividing strains of each genotype were split and used to isolate RNA or DNA. (**A**) A map of single copy loci amplified during TERRA measurements (B-E) and Southern blot probes (F). Distance from the centromeric end of the TG repeats is indicated. Primers used in the qRT-PCR and to make the probes are described in S2 Table. (**B–E**) TERRA measured as in Figure 1B. (**F**) Telomeric Southern blots performed after XhoI (top two gels) or HindIII (bottom two gels) digestion. One membrane was first probed for telomere 15L and then Y’+TG sequences (Supplementary Figure S3A). The other membrane was first probed for telomere 10R and finally 13R. Loading control corresponds to SYBR Safe staining of the gel probed for telomeres 10R and 13R.

The effect of each mutation or mutations on TERRA levels across four telomeres, TEL01L, TEL10R, TEL13R and TEL15L, was measured (Figure 2B–E). As has been reported before, the magnitude of the increase in TERRA caused by specific mutations varied across telomeres (26,27). For example, *sir4Δ* caused a ten-fold increase in TERRA from TEL01L but less than a three-fold increase from TEL13R. Interestingly, TERRA levels were similarly increased in *paf1Δ, ctr9Δ* and *sir4Δ* mutants, at all telomeres examined. Furthermore, the effects of *paf1Δ, ctr9Δ* and *sir4Δ* were stronger than the effects of *yku70Δ* or *mre11Δ* at all telomeres. Finally, deletion of *CDC73* in *paf1Δ* or *ctr9Δ* cells did not decrease TERRA levels at any telomere, showing that Cdc73 is not needed for high TERRA levels in these contexts.

It was possible to generate comparatively specific Southern blot probes for TEL10R, TEL13R, TEL15L, but not TEL01L. The effects of each mutation or mutations tested on telomere length across these three telomeres were similar to those observed on Y’ telomeres, using a standard yeast telomere probe (Figure 2F). Overall, there was no strong correlation between telomere length and TERRA levels in *cdc73Δ, paf1Δ, ctr9Δ, mre11Δ, sir4Δ* and *yku70Δ* cells. For example, *cdc73Δ, paf1Δ, ctr9Δ* cells had similarly short TEL10R telomeres, but very different levels of TERRA. Furthermore, *mre11Δ* and *yku70Δ* cells had shorter telomeres than *paf1Δ* and *ctr9Δ* cells but lower TERRA. Thus, our data suggest that short telomere length does not strongly influence TERRA levels.

**Figure 3.**
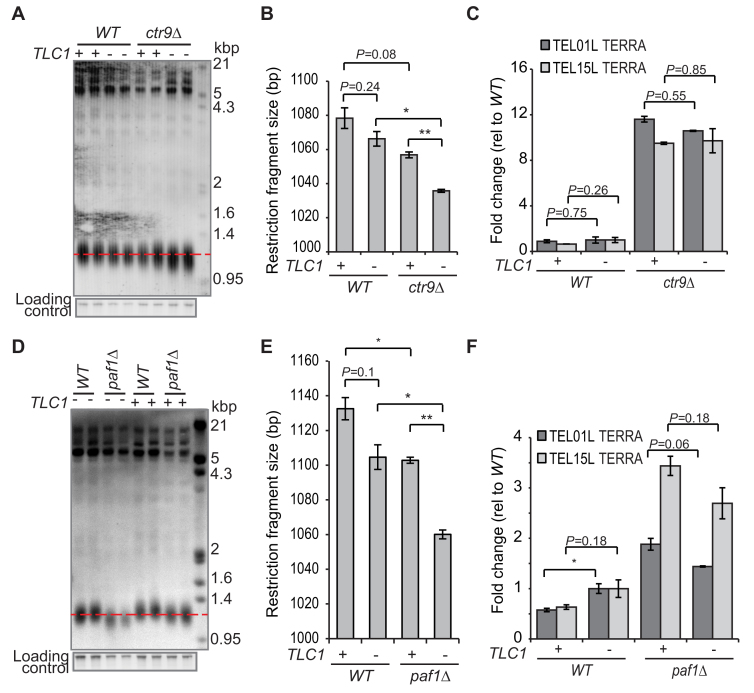
Ctr9 and Paf1 regulate *TERRA* levels independently of telomere length. (**A** and **D**) *ctr9Δ* or *paf1Δ* heterozygous diploid cells were transformed with a centromeric plasmid containing *TLC1* or a vector. After sporulation, pairs of independent *WT, ctr9Δ* (A) and *paf1Δ* (D) haploid cells carrying the *TLC1* or vector plasmid were grown for 72 h at 23°C on –URA liquid media, diluted (1:5000), grown overnight and DNA and RNA were extracted when the cultures reached OD600 of 0.4–0.8. A telomere Southern blot using a Y’ + TG probe (as in Supplementary Figure S3) was performed. (**B** and **E**) ImageJ was used to quantify the restriction fragment sizes of the gels in A or D. (**C** and **F**) TERRA measured as in Figure 1B. TERRA levels were normalized to the mean of the *WT* cells with the vector, using *ACT1* as an internal control. Statistical analyses used the two-tailed unpaired *t*-test (*P < 0.05, **P < 0.01 and ***P < 0.001).

Our data suggest that Paf1 and Ctr9 repress TERRA independently of their effect on telomere length, yet previous experiments have shown that telomere length affects TERRA and vice versa. To further test the hypothesis that Paf1 and Ctr9 are required to maintain low TERRA levels independently of telomere length, we created *ctr9Δ* and *paf1Δ* mutants with comparatively normal telomere lengths. Consistent with what has been reported before, overexpression of *TLC1* largely rescued telomere length defects of *ctr9Δ* and *paf1Δ* cells (Figure 3A, B, D, E) (13). Importantly, *ctr9Δ* and *paf1Δ* cells with comparatively normal telomere length, had high TERRA levels (Figure 3C, F). We conclude that Ctr9 and Paf1 regulate TERRA independently of their effects on telomere length.

### Paf1 and Ctr9 affect TERRA independently of Sir4

**Figure 4.**
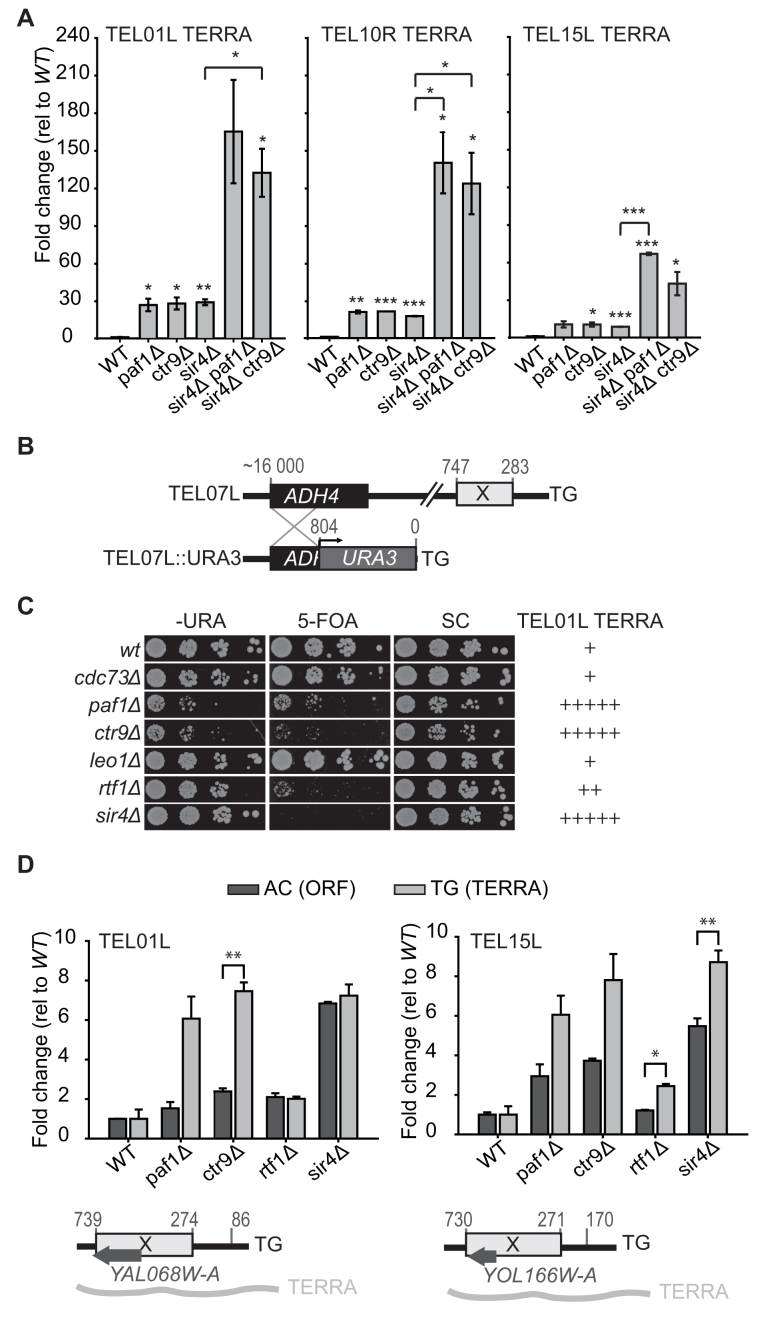
Paf1 and Ctr9 affect telomere silencing but repress TERRA independently of the Sir complex. (**A**) qRT-PCR of TERRA as described in Figure 1B. (**B**) Map of *URA3* integrated at the *ADH4* locus and used to measure telomere silencing (51,52). (**C**) Silencing was measured by comparing growth on plates lacking uracil (-URA), containing 5-Fluoro-orotic acid (5-FOA) or synthetic complete media (SC), at 23°C. Additional strains and temperatures are shown in Supplementary Figure S4. On the right, the relative effects of mutations on TERRA from telomere 1L (from Figure 1B and Figure 2B), are indicated. (**D**) qRT-PCR of RNAs at *YAL068W-A* (telomere 1L) and *YOL166W-A* (telomere 15L) loci, was performed as described in Figure 1B. Dark grey bars represent RNA transcribed in the direction from the telomere towards the centromere (ORF expression) and light grey bars represent transcription in the opposite direction (TERRA). A scheme with the position of each ORF relative to the telomere end and the X element is underneath each graph. See Supplementary Figure S5 for a more detailed explanation of the RNA measurements from TEL01L. Statistical analyses used two-tailed unpaired *t*-test (*P < 0.05, **P < 0.01 and ***P < 0.001).

Since the effects of Paf1 and Ctr9 repressing TERRA were as strong as Sir4 we speculated that Paf1/Ctr9 and Sir4 function in the same pathway of TERRA regulation. To test this, TERRA levels in *sir4Δ paf1Δ* and *sir4Δ ctr9Δ* double mutants were assessed. Interestingly, the *sir4Δ paf1Δ* and *sir4Δ ctr9Δ* double mutants had 4 to 8 times higher TERRA than the corresponding single mutants from TEL01L, TEL10R and TEL15L (Figure 4A). We conclude that Paf1 and Ctr9 maintain low TERRA levels by a mechanism that is largely distinct to Sir4.

Paf1 and Rtf1 have been reported, like Sir4, to help silence telomeric transcription (7). Therefore, it was plausible that the PAF1 complex could contribute to low TERRA by silencing telomeres through a parallel pathway to the Sir complex (affecting histone methylation levels for instance). To test this, *URA3* expression was measured near a truncated 7L telomere, often used to measure telomeric silencing (Figure 4B) (51,52). *paf1Δ, ctr9Δ, rtf1Δ* and *sir4Δ* cells were unable to silence 7L while *cdc73Δ* and *leo1Δ* cells were proficient at silencing (judged by growth on 5-FOA plates) (Figure 4C and Supplementary Figure S4). These results confirm and extend those previously reported (7). Curiously, the *sir4Δ* mutation seemed to suppress the poor growth fitness phenotype of *paf1Δ* and *ctr9Δ* cells, particularly at 34°C (Supplementary Figure S4). We do not understand this observation but perhaps Sir4-regulated transcripts contribute to the fitness of *paf1Δ* and *ctr9Δ* mutants at high temperatures. Importantly, there was a clear lack of correlation between telomere silencing in this assay and TERRA expression (Figure 4C). In particular, *rtf1Δ* cells were perhaps the most defective at silencing the truncated 7L telomere (most similar to *sir4Δ* cells), but showed very small effects on TERRA levels (Figure 1B, C). These silencing assays suggested that PAF1 complex components affected TERRA and silencing at least partially by different mechanisms.

We were concerned that the lack of correlation between 7L silencing and TERRA levels was some type of artefact of the artificial telomere used to measure silencing. The truncated 7L telomere has lost a total of 16kb of telomeric DNA, including the X repeat, found on all normal telomeres (Figure 4B). Furthermore, it is clear that *paf1Δ* and *ctr9Δ* mutations strongly affect TERRA from X-only telomeres (Figure 2) and telomere silencing is centred on the X repeat (53,54). To more directly investigate the effects of Paf1, Ctr9 and Rtf1, TERRA and telomere silencing were measured at two natural telomeres. For this, TERRA transcripts and transcripts from the opposite strand (ORF transcripts) of TEL01L and TEL15L were measured. Interestingly, and most clearly at the TEL01L, the mutations de-repress TERRA levels to a greater extent than ORF transcript levels (Figure 4D). There was also a small increase of the ORF transcript in *paf1Δ* and *ctr9Δ* mutants, consistent with a defect in telomere silencing. *rtf1Δ*, in contrast, affected transcripts from both strands of TEL01L similarly, consistent with *rtf1Δ* mutants showing a general telomere silencing defect. *sir4Δ*, like *rtf1Δ*, affected transcripts from both strands of TEL01L similarly, consistent with the *sir4Δ* mutation causing a general telomere silencing defect, as expected. The silencing defect in *sir4Δ* mutants was greater than in *rtf1Δ* mutants, consistent with truncated chromosome 7L silencing assays (Figure 4C, D). Analysis of transcription from TEL15L revealed a broadly similar pattern, although at this telomere it seemed that all mutations affected TERRA more than transcription from the opposite strand. The observation that Paf1 and Ctr9 show differential effects on telomeric RNA levels, depending on which strand is transcribed, shows that Paf1 and Ctr9 are not silencing all transcription near telomeres and suggests that their effects on TERRA are mainly via a different mechanism.

### Paf1 promotes TERRA degradation

**Figure 5.**
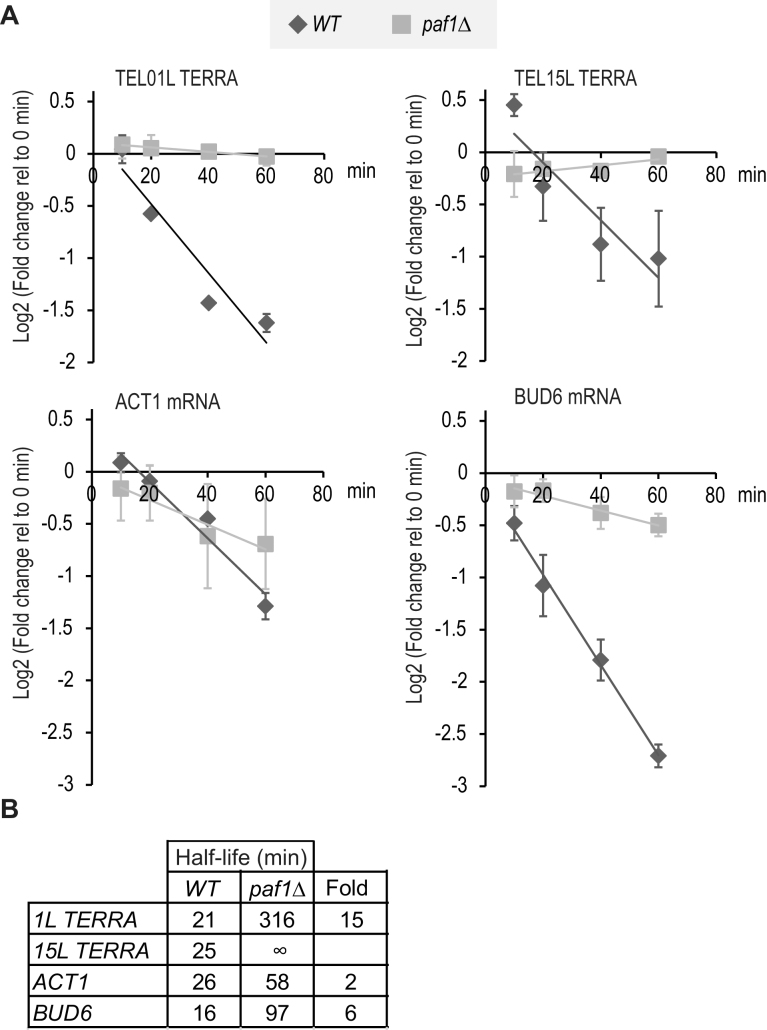
Paf1 stabilizes TERRA and other RNAs. Two independent *paf1Δ* strains, and a single *WT* strain (DLY11213/MP0455) cultured twice, carrying the two fusion proteins Rpb1*FRB (Rpb1, part of RNA pol II; FRB, domain of human mTOR) and Rpl13a-FKBP12 (Rpl13a, ribosomal protein; FKBP12, FK-506 binding protein) were treated with rapamycin. Rapamycin forms a ternary complex with FRB and FKBP12, leading to the removal of Rpb1 from the nucleus [34]. RNA levels at different time points after rapamycin addition were used to calculate half-life. (**A**) qRT-PCR of 01L and 15L TERRA and *ACT1, BUD6* after addition of rapamycin (each replicate and DMSO treatments are in Supplementary Figure S6). Data from two independent strains are shown and the error bars represent average deviation. (**B**) Half-life (min) was calculated as described in Materials and Methods using the slope of the lines in A. ‘∞’ is shown when no RNA decrease was observed over 60 min (a positive slope was observed). Fold changes (Fold) of *paf1Δ* strains relative to *WT* are indicated.

Since Paf1 and Ctr9 can regulate TERRA independently of the Sir complex and telomere silencing, we hypothesized that Paf1 and Ctr9 affected TERRA degradation. To test this, the anchor-away system was used to remove the largest RNA polymerase II subunit (Rpb1) from the nucleus upon rapamycin addition. This stops transcription and permits measurement of RNA decay rates (43). We observed that the half-life of TERRA is similar to that of *ACT1* and *BUD6*, in wild type cells, as reported by others (Figure 5) (26,55). The effect of *paf1Δ* on TERRA decay was clear, dramatically increasing TERRA half-life (Figure 5, Supplementary Figure S6). Interestingly, *ACT1* and *BUD6* RNA half-lives also increased, but much less, 2–6-fold, suggesting that Paf1 stimulates general RNA degradation and TERRA degradation in particular.

During anchor-away strain construction we noticed that *paf1Δ* cells were particularly unfit in the genetic background required for these experiments. Therefore, to confirm the *paf1Δ* effect on RNA stability, we used two complementary approaches (56). 1,10-Phenanthroline, a metal chelator, and thiolutin, a sulfur-containing antibiotic, have been previously used to determine mRNA half-lives (57–59). After 1,10-phenanthroline treatment TERRA from TEL01L and TEL15L in *paf1Δ* mutants was higher than in wild type and *sir4Δ* mutants, consistent with Paf1 playing a role in TERRA degradation (Supplementary Figure S7A). Interestingly, and consistent with the anchor-away experiments, *paf1Δ* cells contained higher levels of *ACT1* and *BUD6* mRNA consistent with a role for Paf1 in degradation of these transcripts too. After thiolutin treatment, TERRA from TEL01L and TEL15L was stabilized in *paf1Δ* cells in comparison to *ACT1* RNA (Supplementary Figure S7B). In summary, three different experimental approaches to measure RNA decay support the view that Paf1 and Ctr9 promote TERRA degradation.

### Paf1, Rat1 and the TRAMP complex independently reduce TERRA levels

**Figure 6.**
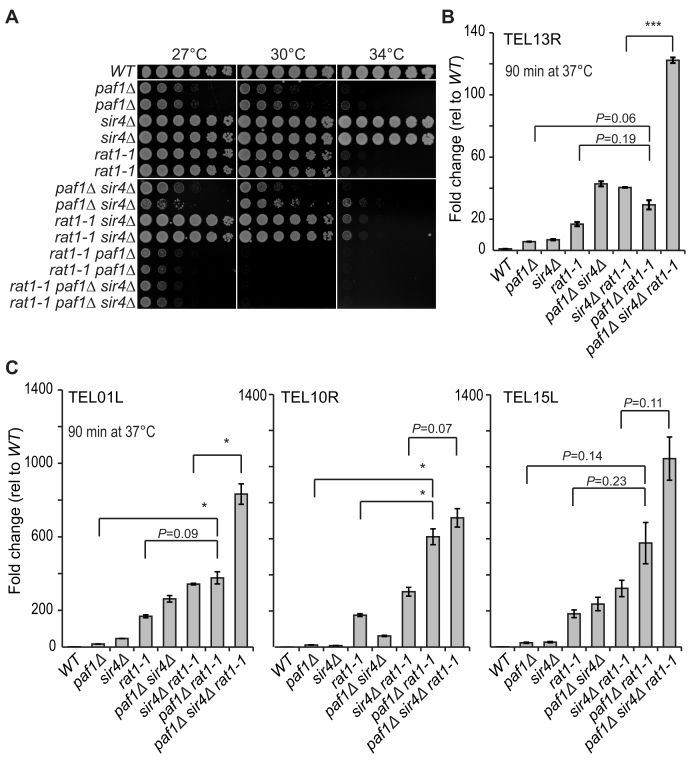
Evidence that Paf1 regulates TERRA levels independently of Rat1. Pairs of independent mutant strains, and a single *WT* strain were cultured overnight. (**A**) Spot tests as described in Figure 1D were performed. (**B** and **C**). Cell cultures used for A were diluted (1:5000) and when the OD600 was between 0.5 and 0.7 the temperature was shifted from 23°C to 37°C for 90 min (31). TERRA measurements were performed as described in Figure 1B. The average of two independent strains was normalized relative to the *WT* strain. Error bars represent average deviation. Statistical analyses used the two-tailed unpaired T test (*P < 0.05 and ***P < 0.001).

Since Paf1 (and Ctr9) promote TERRA degradation it was possible that Paf1 affected the Rat1 or nuclear exosome pathways of degradation, or both (18,19,26,31). *RAT1* is an essential gene and therefore to test interactions with Rat1, a temperature sensitive *rat1–1* allele was combined with *paf1Δ* and/or *sir4Δ* mutations and cell fitness and TERRA levels measured. Interestingly, *paf1Δ* enhances the ts phenotype caused by *rat1–1*, suggesting, perhaps, that Paf1 facilitates Rat1 activity (Figure 6A). Furthermore, others have reported that *paf1Δ* is synthetically sick with mutations affecting RNA stability, suggesting Paf1 facilitates RNA degradation (9). *sir4Δ*, in contrast, shows little synthetic fitness interaction with *rat1–1*. Since *paf1Δ* and *sir4Δ* mutations cause a similar increase in TERRA (Figure 6B), but only the *paf1Δ* mutation is synthetically sick with *rat1–1*, we conclude that synthetic sickness is not due simply to the very high TERRA levels.

A previously established protocol was used to explore the interactions between Rat1 and Paf1 on TERRA levels. Cells were cultured for 90 min at 37°C to inactivate Rat1–1 (31). Under these conditions *paf1Δ* and *sir4Δ* mutations affected TERRA similarly but less than *rat1–1* (Figure 6B, C). Previous data show that Rat1 and the Sir complex affect TERRA through independent pathways (26) and our results confirm this (Figure 6B, C). The data also show that *rat1–1* strongly increases TERRA levels in *paf1Δ* and *paf1Δ sir4Δ* cells. Overall these data are consistent with the idea that Paf1/Ctr9, Sir4 and Rat1 contribute independently to regulate TERRA levels.

**Figure 7.**
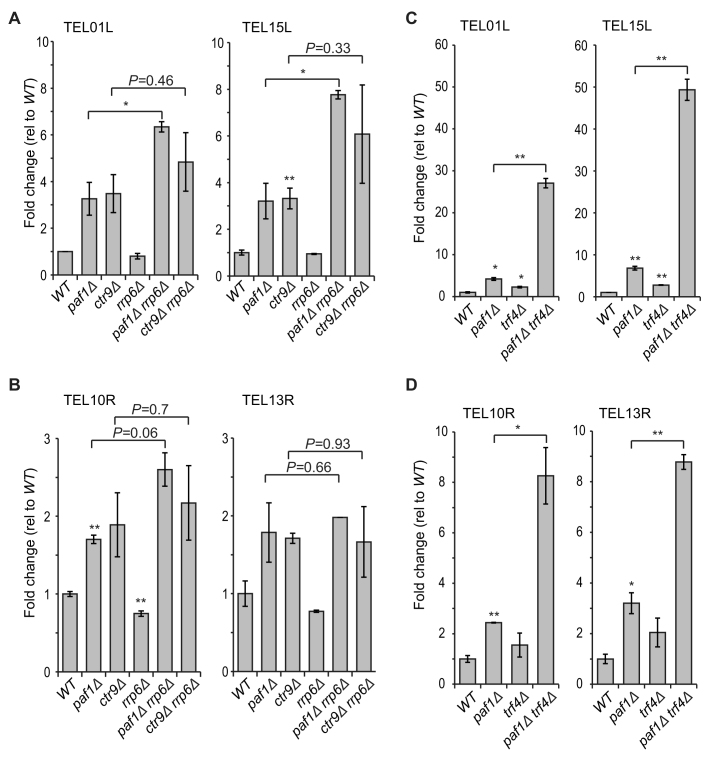
Paf1 regulates TERRA independently of Trf4. (**A–D**) TERRA measurements were performed on two independent strains of each genotype as described in Figure 1B and the mean is shown. Error bars represent average deviation. Statistical analyses used the two-tailed unpaired T test (*P < 0.05, **P < 0.01 and ***P < 0.001).

There is evidence that the TRAMP complex facilitates TERRA degradation in *rat1–1* mutants, although no increase in TERRA levels was seen in *trf4Δ* (defective in the TRAMP complex) or *rrp6Δ* (defective in the nuclear exosome) single mutants (31). To test whether Paf1 and Ctr9 regulate TERRA through the TRAMP/exosome pathway *rrp6Δ* and *trf4Δ* were deleted in *ctr9Δ* and *paf1Δ* mutants. As previously reported, *rrp6Δ* cells did not show increased TERRA and we did not see any synergistic increase in TERRA levels of *paf1Δ rrp6Δ* or *ctr9Δ rrp6Δ* cells (Figure 7A, B). This suggests that the nuclear exosome has a comparatively minor role in TERRA degradation. Interestingly, a small but significant increase in TERRA levels was observed in *trf4Δ* cells (Figure 7C, D). Consistent with the idea that the TRAMP complex affects TERRA independently of Paf1, *paf1Δ trf4Δ* cells have much higher levels of TERRA than *paf1Δ* or *trf4Δ* cells (Figure 7C, D). We conclude that the PAF1 complex regulates TERRA independently of Trf4 (and the TRAMP complex). Additionally, our data suggest that the TRAMP complex could promote Rrp6(exosome)-independent TERRA degradation. Overall the data suggest that Paf1 and Ctr9 can affect TERRA levels independently of the known, Rat1- and Trf4-dependent, pathways. One explanation for these data is that the Paf1 complex helps both Rat1 and Trf4 pathways to degrade TERRA.

### Paf1 and Ctr9 localise at telomeres

**Figure 8.**
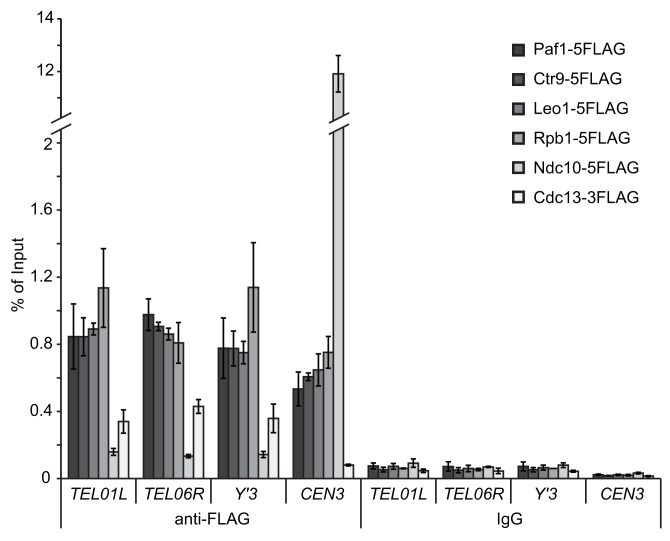
The PAF1 complex is present at telomeres. ChIP analysis of FLAG-tagged proteins at telomeric and centromeric loci (TEL01L, TEL06R, Y’3 and *CEN3*). ChIP was performed using either an anti-FLAG antibody or an anti-mouse IgG as a control. The amount of each locus precipitated is plotted as percentage of input. Two independent strains for each of genotype were processed except for the Cdc13 and Ndc10 FLAG tagged strains where in each case a single strain was cultured twice. The mean is shown and error bars represent average deviation. Protein molecular weight/levels, telomere length and cell fitness of the new epitope tagged strains are shown in Supplementary Figure S8.

One hypothesis for how Paf1 and Ctr9 affect TERRA levels more than Cdc73, Leo1 or Rtf1, is that these are the only two components that travel with RNA pol II to transcribe TERRA. To test this hypothesis, we performed ChIP analysis on Paf1, Ctr9 and Leo1. As controls, we also performed ChIP on Rpb1, a subunit of RNA pol II (since the PAF1 complex associates with RNA pol II), Cdc13 (a telomere binding protein) and Ndc10 (a centromere binding protein). As expected, Cdc13 strongly associated with telomeric DNA while Ndc10 strongly associated with centromeric DNA, showing specificity in these experiments (Figure 8). In contrast, we found that Paf1, Ctr9, Leo1 and Rpb1 associate both with telomeric (TEL01L, TEL06R, Y’3) and centromeric (*CEN3*) DNA (Figure 8). These data are consistent with the idea that Paf1, Ctr9 and Leo1 are part of a single TEF complex that transcribes telomeres and centromeres and that the existence of different Paf1 sub-complexes is not the explanation for the different effects of Paf1/Ctr9 versus Leo1 on TERRA levels.

## DISCUSSION

The Paf1 complex is conserved, binds RNA polymerase II, and affects the abundance of many transcripts. Previous experiments in yeast have connected components of the PAF1 complex with telomere functions. Specifically, Paf1, Ctr9 and Cdc73 have been reported to contribute to telomerase template RNA expression and normal telomere length regulation (13). Here, we report that, of these, Paf1 and Ctr9, but not Cdc73, are required to maintain low TERRA levels (a conserved non-coding telomeric RNA found across eukaryotes from yeast to mammalian cells). Interestingly, loss of Paf1 and Ctr9 also reduces cell fitness more than loss of Cdc73. These observations show that high TERRA correlates better with poor cell fitness across cells lacking Cdc73, Paf1 or Ctr9, than low TLC1 levels.

There is a correlation between high TERRA levels and poor fitness of *paf1Δ* and *ctr9Δ* mutants, suggesting that TERRA or other dysregulated transcripts might be toxic. One mechanism that might explain why high TERRA itself, is toxic, is that in combination with loss of Paf1/Ctr9 TERRA is particularly harmful. This could be due to the role of the Paf1 complex in resolving transcription/replication fork collisions. *paf1Δ* cells demonstrate slow replication-fork progression and increased transcription–replication fork collisions, particularly when RNA and DNA polymerases are travelling in the same direction (10). At the telomere, RNA and DNA polymerases travel in the same direction, toward the chromosome end, and this, in combination with high levels of TERRA, and therefore increased levels of RNA:DNA hybrids may be a particular issue for *paf1Δ* and *ctr9Δ* cells.

There are many reports that telomere length affects TERRA and vice versa. However, Cdc73, Paf1 and Ctr9 all have similar effects on telomere length but affect TERRA to very different extents, showing that these proteins do not seem to affect TERRA via affecting telomere length. Consistent with this, when telomere length was normalized in cells lacking Ctr9 or Paf1, TERRA levels remained high. Thus, at least across *cdc73Δ, paf1Δ* and *ctr9Δ* cells there is not a good correlation between TERRA and telomere length, suggesting Paf1 and Ctr9 have more direct effects on TERRA.

How the PAF1 complex affects TERRA is still not completely clear. The PAF1 complex affects the levels of many transcripts, both positively and negatively, and therefore it is possible that Paf1/Ctr9 might affect TERRA via a number of mechanisms. *paf1Δ* strongly increased TERRA levels in *sir4Δ, rat1–1* and *trf4Δ* strains. On this basis, we cannot assign Paf1/Ctr9 to just one of the three major pathways of TERRA regulation (Sir4, Rat1 or Trf4-dependent). The most logical explanation for these results is that Paf1 and Ctr9 affect TERRA in many ways, transcriptionally and post-transcriptionally, for example affecting Sir4, Rat1 or Trf4-dependent pathways of TERRA regulation.

Our data, and recent structural data, are consistent with a model where the entire PAF1 complex is stably associated with Pol II and reaches the telomere (10). We suggest that the Paf1 complex recruits other factors, such as nucleases and histone methylases, to perform subunit-specific functions and that loss of Paf1/Ctr9, the scaffolding proteins, leads to the disruption of most PAF1-related functions and that more than one of these functions affect TERRA levels. Mutations in *CDC73* and *CTR9* have been identified in cancer and telomere function is often affected in cancer. It seems plausible that one of the ways that the PAF1 complex affects human cancer is by affecting TERRA.

## Supplementary Material

Supplementary Data
