# Supplementary material for: Paf1 and Ctr9, core components of the PAF1 complex, maintain low levels of telomeric repeat containing RNA
Source: Nucleic Acids Res. 2017 Nov 14;46(2):621–34. doi: 10.1093/nar/gkx1131 (PMC5778495; doi:10.1093/nar/gkx1131)

Paf1 and Ctr9, core components of the PAF1 complex, maintain low levels of telomeric repeat containing RNA

Joana Rodrigues and David Lydall

SUPPLEMENTARY FIGURES AND TABLES

S1 Fig. Cdc73, Paf1 and Ctr9 affect *TLC1* RNA levels.
One-step qRT-PCR analysis of *TLC1* RNA expression levels. RNA from two strains of each genotype. Each value was normalized to the levels of *BUD6* mRNA and is indicated as fold change over *WT* (Addinall et al. 2011). The “error” bars indicate the average deviation. Statistical analyses used the two-tailed unpaired T test (***P*<0.01 and ****P*<0.001).

S2 Fig. Paf1 and Ctr9 similarly regulate polyadenylated and non-polyadenylated TERRA.
(A) Scheme showing the method used to determine the levels of total TERRA and polyadenylated TERRA. Two reverse transcription reactions were performed: i) using a CA-rich primer (to measure total TERRA) and an *ACT1* reverse primer; or ii) using an Oligo dT15 reverse primer (to measure polyadenylated TERRA). In both cases TERRA levels were measured from specific telomeres by qPCR using telomere-specific forward primers (TEL01L or TEL15L) and the CA-rich reverse primer. *ACT1* was used as an internal control. **(B)** Relative amounts of non-polyadenylated TERRA and polyadenylated TERRA from TEL01L and TEL15L are shown. Values for non-polyadenylated TERRA were calculated by subtracting the values of the polyadenylated TERRA from the total TERRA (D). The fold change relative to *WT* values is shown. The RNA used is the same as in Figure 6A,B. Statistical analyses used two-tailed unpaired T test (***P*<0.01 and ****P*<0.001) and compare the mutants to *WT.* **(C)** Fraction of polyadenylated TERRA calculated from the values in D (polyadenylated TERRA/Total TERRA). **(D)** Raw data showing the average CTs (of the technical triplicates) and the 2^-ΔCT values.

S3 Fig. Cdc73, Paf1 and Ctr9 affect telomere length.
(A) The locations of a telomere Southern blot probe and XhoI sites. The probe recognizes both Y’ and TG sequences. Distance from the centromeric end of the TG repeats is indicated. Adapted from (Maringele and Lydall 2004). **(B)** A representative telomere Southern blot using the probe in A. All lanes were from a single gel, that was cropped for presentation purposes. ImageJ and statistical analysis of this blot and others performed independently using a two tailed T test showed significant differences in telomere length to *WT* for *cdc73Δ* (P = 0.0002), *paf1Δ* (P = 0.0084) and *ctr9Δ* (P = 0.018) but not for *rtf1Δ* (P = 0.26) or *leo1Δ* (P = 0.088).

S4 Fig. PAF1 components and telomere silencing.
More extensive data from the silencing experiment performed in Figure 4C.

S5 Fig. Strand specific RNA measurements.
(A) To compare transcription of both strands of TEL01L, a pair of independent cDNA synthesis reactions were performed using primers m4273 or m4274. m4273 stimulates cDNA synthesis form TERRA, and m4274 cDNA from the *YAL068W-A* transcript. qPCR was then performed using m4273 and m4274.

S6 Fig. Paf1 promotes RNA degradation.
The data used to generate Figure 5. Two-step qRT-PCR of *ACT1*, *BUD6* and *TERRA* from 2 different telomeres (1L and 15L), was performed at the times indicated after rapamycin or DMSO addition. Experiments a and b, performed on different days, are shown. RNA levels were normalized for the amount of 7S RNA (Iglesias et al. 2011). The fold change from the 0 min time point was calculated and plotted. The linear regression lines through each set of data is shown, starting from the 10 min time point for the rapamycin treated samples (Iglesias et al. 2011). The equations at the top or each plot refer to rapamycin treated samples and are of the form y = mx + c, where m is the slope of the curve.

S7 Fig. Paf1 and Ctr9 stabilize TERRA.
(A and B) Transcription shutoff using 1,10-phenanthroline (A) or thiolutin (B). RNA levels at different time points after the drug treatment were measured and normalized to the levels of 7S RNA. Data are expressed as a percentage of the amounts present before the drug treatment (time point 0 min). In B two independent experiments were performed, and the mean is shown. Error bars represent average deviation.

S8 Fig. FLAG epitope on PAF1 complex components does not affect telomere length or fitness.
(A) Western-blot using an anti-FLAG antibody (F3165) was performed as described in (Morin et al. 2008) on pairs of epitope strains, except for Ndc10, to show that tagged proteins are stable and of the expected molecular weight. **(B)** Telomere Southern blot was performed on the strains in A, as described in Figure 2F (using the Y'+TG probe). **(C)** Spot tests were performed on the strains in A, as described in Figure 1D, except that the cultures were grown to saturation at 30°C.

Table S1 – Yeast strains used in this study.

DLY	Relevant genotype	Origin
8460	*MATa ade2-1 trp1-1 can1-100 leu2-3,112 his3-11,15 ura3 GAL+ psi+ ssd1-d2 RAD5*	Rodney Rothstein
3001	*MATalpha ade2-1 trp1-1 can1-100 leu2-3,112 his3-11,15 ura3 GAL+ psi+ ssd1-d2 RAD5*	Rodney Rothstein
8490	*MATa ade2-1 trp1-1 can1-100 leu2-3,112 his3-11,15 ura3 GAL+ psi+ ssd1-d2 RAD5 cdc73::KANMX*	This study
8491	*MATalpha ade2-1 trp1-1 can1-100 leu2-3,112 his3-11,15 ura3 GAL+ psi+ ssd1-d2 RAD5 cdc73::KANMX*	This study
8757	*MATa ade2-1 trp1-1 can1-100 leu2-3,112 his3-11,15 ura3 GAL+ psi+ ssd1-d2 RAD5 paf1::KANMX*	This study
8758	*MATalpha ade2-1 trp1-1 can1-100 leu2-3,112 his3-11,15 ura3 GAL+ psi+ ssd1-d2 RAD5 paf1::KANMX*	This study
8751	*MATa ade2-1 trp1-1 can1-100 leu2-3,112 his3-11,15 ura3 GAL+ psi+ ssd1-d2 RAD5 ctr9::KANMX*	This study
8752	*MATalpha ade2-1 trp1-1 can1-100 leu2-3,112 his3-11,15 ura3 GAL+ psi+ ssd1-d2 RAD5 ctr9::KANMX*	This study
1412	*MATa ade2-1 trp1-1 can1-100 leu2-3,112 his3-11,15 ura3 GAL+ psi+ ssd1-d2 RAD5 yku70::HIS3*	Lydall lab
1366	*MATalpha ade2-1 trp1-1 can1-100 leu2-3,112 his3-11,15 ura3 GAL+ psi+ ssd1-d2 RAD5 yku70::HIS3*	Lydall lab
2041	*MATalpha ade2-1 trp1-1 can1-100 leu2-3,112 his3-11,15 ura3 GAL+ psi+ ssd1-d2 RAD5 mre11::hisG::URA3*	Lydall lab
4457	*MATalpha ade2-1 trp1-1 can1-100 leu2-3,112 his3-11,15 ura3 GAL+ psi+ ssd1-d2 RAD5 mre11::URA3*	Lydall lab
5692	*MATa ade2-1 trp1-1 can1-100 leu2-3,112 his3-11,15 ura3 GAL+ psi+ ssd1-d2 RAD5 sir4::HIS3 hml::leu2::URA3*	Lydall lab
5693	*MATa ade2-1 trp1-1 can1-100 leu2-3,112 his3-11,15 ura3 GAL+ psi+ ssd1-d2 RAD5 sir4::HIS3 hml::leu2::URA3*	Lydall lab
10959	*MATa ade2-1 trp1-1 can1-100 leu2-3,112 his3-11,15 ura3 GAL+ psi+ ssd1-d2 RAD5 cdc73::HPH paf1::KANMX*	This study
10960	*MATa ade2-1 trp1-1 can1-100 leu2-3,112 his3-11,15 ura3 GAL+ psi+ ssd1-d2 RAD5 cdc73::HPH paf1::KANMX*	This study
10961	*MATalpha ade2-1 trp1-1 can1-100 leu2-3,112 his3-11,15 ura3 GAL+ psi+ ssd1-d2 RAD5 cdc73::HPH ctr9::KANMX*	This study
10962	*MATalpha ade2-1 trp1-1 can1-100 leu2-3,112 his3-11,15 ura3 GAL+ psi+ ssd1-d2 RAD5 cdc73::HPH ctr9::KANMX*	This study
8594	*MATa ade2-1 trp1-1 can1-100 leu2-3,112 his3-11,15 ura3 GAL+ psi+ ssd1-d2 RAD5 yku70::HIS3 cdc73::KANMX*	This study
8595	*MATalpha ade2-1 trp1-1 can1-100 leu2-3,112 his3-11,15 ura3 GAL+ psi+ ssd1-d2 RAD5 yku70::HIS3 cdc73::KANMX*	This study
8702	*MATa ade2-1 trp1-1 can1-100 leu2-3,112 his3-11,15 ura3 GAL+ psi+ ssd1-d2 RAD5 yku80::HIS3 cdc73::KANMX*	This study
8703	*MATalpha ade2-1 trp1-1 can1-100 leu2-3,112 his3-11,15 ura3 GAL+ psi+ ssd1-d2 RAD5 yku80::HIS3 cdc73::KANMX*	This study
8736	*MATa ade2-1 trp1-1 can1-100 leu2-3,112 his3-11,15 ura3 GAL+ psi+ ssd1-d2 RAD5 leo1::KANMX*	This study
8737	*MATa ade2-1 trp1-1 can1-100 leu2-3,112 his3-11,15 ura3 GAL+ psi+ ssd1-d2 RAD5 leo1::KANMX*	This study
8743	*MATa ade2-1 trp1-1 can1-100 leu2-3,112 his3-11,15 ura3 GAL+ psi+ ssd1-d2 RAD5 rtf1::KANMX*	This study
8744	*MATalpha ade2-1 trp1-1 can1-100 leu2-3,112 his3-11,15 ura3 GAL+ psi+ ssd1-d2 RAD5 rtf1::KANMX*	This study
2146	*MATa ade2-1 trp1-1 can1-100 leu2-3,112 his3-11,15 ura3 GAL+ psi+ ssd1-d2 RAD5 tlc1::HIS3*	Lydall lab
2147	*MATalpha ade2-1 trp1-1 can1-100 leu2-3,112 his3-11,15 ura3 GAL+ psi+ ssd1-d2 RAD5 tlc1::HIS3*	Lydall lab
2311	*MATa ade2-1 trp1-1 can1-100 leu2-3,112 his3-11,15 ura3 GAL+ psi+ ssd1-d2 RAD5 telVII-L-URA3*	Dr. Eric Gilson
11154	*MATa ade2-1 trp1-1 can1-100 leu2-3,112 his3-11,15 ura3 GAL+ psi+ ssd1-d2 RAD5 sir4::HIS3 paf1::KANMX hml::leu2::URA3*	This study

11155	*MATalpha ade2-1 trp1-1 can1-100 leu2-3,112 his3-11,15 ura3 GAL+ psi+ ssd1-d2 RAD5 sir4::HIS3 paf1::KANMX (sterile)*	This study
11156	*MAT? ade2-1 trp1-1 can1-100 leu2-3,112 his3-11,15 ura3 GAL+ psi+ ssd1-d2 RAD5 sir4::HIS3 ctr9::KANMX (sterile)*	This study
11157	*MATalpha ade2-1 trp1-1 can1-100 leu2-3,112 his3-11,15 ura3 GAL+ psi+ ssd1-d2 RAD5 sir4::HIS3 ctr9::KANMX hml::leu2::URA3 (sterile)*	This study
11158	*MATa ade2-1 trp1-1 can1-100 leu2-3,112 his3-11,15 ura3 GAL+ psi+ ssd1-d2 rad5-535 telVII-L URA3 cdc73::KANMX*	This study
11159	*MATa ade2-1 trp1-1 can1-100 leu2-3,112 his3-11,15 ura3 GAL+ psi+ ssd1-d2 rad5-535 telVII-L URA3 cdc73::KANMX*	This study
11161	*MATa ade2-1 trp1-1 can1-100 leu2-3,112 his3-11,15 ura3 GAL+ psi+ ssd1-d2 rad5-535 telVII-L URA3 paf1::KANMX*	This study
11162	*MATa ade2-1 trp1-1 can1-100 leu2-3,112 his3-11,15 ura3 GAL+ psi+ ssd1-d2 rad5-535 telVII-L URA3 paf1::KANMX*	This study
11164	*MATa ade2-1 trp1-1 can1-100 leu2-3,112 his3-11,15 ura3 GAL+ psi+ ssd1-d2 rad5-535 telVII-L URA3 ctr9::KANMX*	This study
11165	*MATa ade2-1 trp1-1 can1-100 leu2-3,112 his3-11,15 ura3 GAL+ psi+ ssd1-d2 rad5-535 telVII-L URA3 ctr9::KANMX*	This study
11167	*MATa ade2-1 trp1-1 can1-100 leu2-3,112 his3-11,15 ura3 GAL+ psi+ ssd1-d2 rad5-535 telVII-L URA3 leo1::KANMX*	This study
11168	*MATa ade2-1 trp1-1 can1-100 leu2-3,112 his3-11,15 ura3 GAL+ psi+ ssd1-d2 rad5-535 telVII-L URA3 leo1::KANMX*	This study
11169	*MATa ade2-1 trp1-1 can1-100 leu2-3,112 his3-11,15 ura3 GAL+ psi+ ssd1-d2 rad5-535 telVII-L URA3 rtf1::KANMX*	This study
11170	*MATa ade2-1 trp1-1 can1-100 leu2-3,112 his3-11,15 ura3 GAL+ psi+ ssd1-d2 rad5-535 telVII-L URA3 rtf1::KANMX*	This study
11213	*MATalpha tor1-1 fpr1::NAT RPL13A-2*FKBP12::TRP1 Rpb1-FRB-kanMX6*	(Haruki et al. 2008), D. Manolis Papamichos-Chronakis (YM0455)
11218	*MATalpha tor1-1 fpr1::NAT RPL13A-2*FKBP12::TRP1 Rpb1-FRB-kanMX6 paf1::HPH*	This study
11232	*MATalpha tor1-1 fpr1::NAT RPL13A-2*FKBP12::TRP1 Rpb1-FRB-kanMX6 paf1::HPH*	This study
11791	*MATalpha ade2-1 trp1-1 can1-100 leu2-3,112 his3-11,15 ura3 GAL+ psi+ ssd1-d2 RAD5 rat1-1:KANMX*	This study*
11810	*MATa ade2-1 trp1-1 can1-100 leu2-3,112 his3-11,15 ura3 GAL+ psi+ ssd1-d2 RAD5 paf1::HPH exo1::LEU2 sir4::HIS3 hml::leu2::URA3*	This study
11830	*MATalpha ade2-1 trp1-1 can1-100 leu2-3,112 his3-11,15 ura3 GAL+ psi+ ssd1-d2 RAD5 hml::leu2::URA3*	This study
11832	*MATalpha ade2-1 trp1-1 can1-100 leu2-3,112 his3-11,15 ura3 GAL+ psi+ ssd1-d2 RAD5 hml::leu2::URA3 paf1::HPH*	This study
11833	*MATalpha ade2-1 trp1-1 can1-100 leu2-3,112 his3-11,15 ura3 GAL+ psi+ ssd1-d2 RAD5 hml::leu2::URA3 paf1::HPH*	This study
11834	*MATa ade2-1 trp1-1 can1-100 leu2-3,112 his3-11,15 ura3 GAL+ psi+ ssd1-d2 RAD5 hml::leu2::URA3 rat1-1:KANMX*	This study
11835	*MATalpha ade2-1 trp1-1 can1-100 leu2-3,112 his3-11,15 ura3 GAL+ psi+ ssd1-d2 RAD5 hml::leu2::URA3 rat1-1:KANMX*	This study
11836	*MATa ade2-1 trp1-1 can1-100 leu2-3,112 his3-11,15 ura3 GAL+ psi+ ssd1-d2 RAD5 hml::leu2::URA3 rat1-1:KANMX sir4::HIS3*	This study
11837	*MATa ade2-1 trp1-1 can1-100 leu2-3,112 his3-11,15 ura3 GAL+ psi+ ssd1-d2 RAD5 hml::leu2::URA3 rat1-1:KANMX sir4::HIS3*	This study
11838	*MATa ade2-1 trp1-1 can1-100 leu2-3,112 his3-11,15 ura3 GAL+ psi+ ssd1-d2 RAD5 hml::leu2::URA3 rat1-1:KANMX paf1::HPH*	This study
11839	*MATalpha ade2-1 trp1-1 can1-100 leu2-3,112 his3-11,15 ura3 GAL+ psi+ ssd1-d2 RAD5 hml::leu2::URA3 rat1-1:KANMX paf1::HPH*	This study
11840	*MATa ade2-1 trp1-1 can1-100 leu2-3,112 his3-11,15 ura3 GAL+ psi+ ssd1-d2 RAD5 hml::leu2::URA3 rat1-1:KANMX paf1::HPH sir4::HIS3*	This study
11841	*MATa ade2-1 trp1-1 can1-100 leu2-3,112 his3-11,15 ura3 GAL+ psi+ ssd1-d2 RAD5 hml::leu2::URA3 rat1-1:KANMX paf1::HPH sir4::HIS3*	This study
11842	*MATa ade2-1 trp1-1 can1-100 leu2-3,112 his3-11,15 ura3 GAL+ psi+ ssd1-d2 RAD5 hml::leu2::URA3 exo1::LEU2*	This study
11843	*MATa ade2-1 trp1-1 can1-100 leu2-3,112 his3-11,15 ura3 GAL+ psi+ ssd1-d2 RAD5 hml::leu2::URA3 exo1::LEU2*	This study

11844	*MATa ade2-1 trp1-1 can1-100 leu2-3,112 his3-11,15 ura3 GAL+ psi+ ssd1-d2 RAD5 hml::leu2::URA3 exo1::LEU2 paf1::HPH*	This study
11845	*MATalpha ade2-1 trp1-1 can1-100 leu2-3,112 his3-11,15 ura3 GAL+ psi+ ssd1-d2 RAD5 hml::leu2::URA3 exo1::LEU2 paf1::HPH*	This study
11846	*MATa ade2-1 trp1-1 can1-100 leu2-3,112 his3-11,15 ura3 GAL+ psi+ ssd1-d2 RAD5 hml::leu2::URA3 exo1::LEU2 sir4::HIS3*	This study
11847	*MATa ade2-1 trp1-1 can1-100 leu2-3,112 his3-11,15 ura3 GAL+ psi+ ssd1-d2 RAD5 hml::leu2::URA3 exo1::LEU2 sir4::HIS3*	This study
11848	*MATa ade2-1 trp1-1 can1-100 leu2-3,112 his3-11,15 ura3 GAL+ psi+ ssd1-d2 RAD5 hml::leu2::URA3 exo1::LEU2 sir4::HIS3 paf1::HPH*	This study
11849	*MATa ade2-1 trp1-1 can1-100 leu2-3,112 his3-11,15 ura3 GAL+ psi+ ssd1-d2 RAD5 hml::leu2::URA3 exo1::LEU2 sir4::HIS3 paf1::HPH*	This study
11850	*MATalpha ade2-1 trp1-1 can1-100 leu2-3,112 his3-11,15 ura3 GAL+ psi+ ssd1-d2 RAD5 hml::leu2::URA3 sir4::HIS3 paf1::HPH (sterile)*	This study
12273	*MATalpha ade2-1 trp1-1 can1-100 leu2-3,112 his3-11,15 ura3 GAL+ psi+ ssd1-d2 RAD5 rrp6::HPH*	This study
12274	*MATalpha ade2-1 trp1-1 can1-100 leu2-3,112 his3-11,15 ura3 GAL+ psi+ ssd1-d2 RAD5 rrp6::HPH*	This study
12275	*MATalpha ade2-1 trp1-1 can1-100 leu2-3,112 his3-11,15 ura3 GAL+ psi+ ssd1-d2 RAD5 paf1::KANMX6 rrp6::HPH*	This study
12276	*MATalpha ade2-1 trp1-1 can1-100 leu2-3,112 his3-11,15 ura3 GAL+ psi+ ssd1-d2 RAD5 paf1::KANMX6 rrp6::HPH*	This study
12277	*MATa ade2-1 trp1-1 can1-100 leu2-3,112 his3-11,15 ura3 GAL+ psi+ ssd1-d2 RAD5 ctr9::KANMX6 rrp6::HPH*	This study
12278	*MATalpha ade2-1 trp1-1 can1-100 leu2-3,112 his3-11,15 ura3 GAL+ psi+ ssd1-d2 RAD5 ctr9::KANMX6 rrp6::HPH*	This study
8771	*MATa ade2-1 trp1-1 can1-100 leu2-3,112 his3-11,15 ura3 GAL+ psi+ ssd1-d2 RAD5 CDC13-3FLAG:KANMX6*	Lydall lab
12207	*MATa ade2-1 trp1-1 can1-100 leu2-3,112 his3-11,15 ura3 GAL+ psi+ ssd1-d2 RAD5 PAF1-5FLAG:NAT*	This study
12248	*MATa ade2-1 trp1-1 can1-100 leu2-3,112 his3-11,15 ura3 GAL+ psi+ ssd1-d2 RAD5 PAF1-5FLAG:NAT*	This study
12210	*MATa ade2-1 trp1-1 can1-100 leu2-3,112 his3-11,15 ura3 GAL+ psi+ ssd1-d2 RAD5 CTR9-5FLAG:NAT*	This study
12250	*MATa ade2-1 trp1-1 can1-100 leu2-3,112 his3-11,15 ura3 GAL+ psi+ ssd1-d2 RAD5 CTR9-5FLAG:NAT*	This study
12213	*MATa ade2-1 trp1-1 can1-100 leu2-3,112 his3-11,15 ura3 GAL+ psi+ ssd1-d2 RAD5 LEO1-5FLAG:NAT*	This study
12252	*MATa ade2-1 trp1-1 can1-100 leu2-3,112 his3-11,15 ura3 GAL+ psi+ ssd1-d2 RAD5 LEO1-5FLAG:NAT*	This study
12215	*MATa ade2-1 trp1-1 can1-100 leu2-3,112 his3-11,15 ura3 GAL+ psi+ ssd1-d2 RAD5 RPB1-5FLAG:NAT*	This study
12253	*MATa ade2-1 trp1-1 can1-100 leu2-3,112 his3-11,15 ura3 GAL+ psi+ ssd1-d2 RAD5 RPB1-5FLAG:NAT*	This study
12227	*MATa ade2-1 trp1-1 can1-100 leu2-3,112 his3-11,15 ura3 GAL+ psi+ ssd1-d2 RAD5 NDC10-5FLAG:NAT*	This study
DDY590	*ade2-1 trp1-1 can1-100 leu2-3,112 his3-11,15 ura3 GAL+ psi+ ssd1-d2 RAD5 cdc13-1/CDC13 rad9::HIS3/RAD9 cdc73::KANMX/CDC73*	This study (diploid)
DDY598	*ade2-1 trp1-1 can1-100 leu2-3,112 his3-11,15 ura3 GAL+ psi+ ssd1-d2 RAD5 cdc13-1/CDC13 rad9::HIS3/RAD9 paf1::KANMX/PAF1*	This study (diploid)
DDY1091	*ade2-1 trp1-1 can1-100 leu2-3,112 his3-11,15 ura3 GAL+ psi+ ssd1-d2 RAD5 cdc13-1/CDC13 exo1::LEU2/EXO1 ctr9::KANMX/CTR9*	This study (diploid)
DDY1221	*ade2-1 trp1-1 can1-100 leu2-3,112 his3-11,15 ura3 GAL+ psi+ ssd1-d2 RAD5 cdc13-1/CDC13 exo1::LEU2/EXO1 cdc73::KANMX/CDC73 sir4::HPH/SIR4*	This study (diploid)

*****_rat1-1_ strains were made using a PCR based method to insert the rat1-1:KANMX allele into W303. DNA was extracted from the S288C strain yBL138 (kindly provided by Dr Brian Luke). The *rat1-1:KAN* fragment was amplified by PCR and the PCR product was directly used to transform a wild-type W303 strain (DLY 3001). Temperature sensitivity of the transformants was confirmed by spot test. One of the transformants (DLY11791) was crossed with a strain carrying *sir4Δ paf1Δ exo1Δ hmlΔ* (DLY11810) to generate strains listed above (DLY11830-DLY11850).

Table S2 – List of primers used.

Primer	Annealing region	Sequence	Used for	Primer efficiency*	Ref.
m2864	*CDC73*	AGAATAATAATTTGAGCAAGAAACTGGTGA AAAAATTATGCGGATCCCCGGGTTAATTAA	KO using Longtine plasmids		This study
m2866	CDC73	TTCAATGGCCGAAATACCATTCTTCCGTTT ATCGTATTCAGAATTCGAGCTCGTTTAAAC	KO using Longtine plasmids		This study
m2899	*PAF1*	CAATAGAACAGTGCTCATAATAGTATAAAG GGTCACAATGCGGATCCCCGGGTTAATTA A	KO using Longtine plasmids		This study
m2900	*PAF1*	CAGGTTTAAAATCAATCTCCCTTCACTTCTC AATATTCTAGAATTCGAGCTCGTTTAAAC	KO using Longtine plasmids		This study
m2905	*CTR9*	GTCTGGTCCATTTGTGTTGAGAGCAAGAAA AAAAAACATGCGGATCCCCGGGTTAATTAA	KO using Longtine plasmids		This study
m2906	*CTR9*	TTTCTTTAAAAGTCTTGATTCTAACCCTCGC CTCTTCTTAGAATTCGAGCTCGTTTAAAC	KO using Longtine plasmids		This study
m2887	*LEO1*	AAAGTAATCCAATTAGATATACTGGACTATA ATTAAGATGCGGATCCCCGGGTTAATTAA	KO using Longtine plasmids		This study
m2888	*LEO1*	TGTACATACTAATATATATAAACAAAGTAAC GTCTCCTCTGAATTCGAGCTCGTTTAAAC	KO using Longtine plasmids		This study
m2893	*RTF1*	AATTGTATTGCACTAATTTGTTGAGAGCAC TATAGAAATGCGGATCCCCGGGTTAATTAA	KO using Longtine plasmids		This study
m2894	*RTF1*	AAATATATTTTTACAAACACTGAAATTGTCC TGCCTACTAGAATTCGAGCTCGTTTAAAC	KO using Longtine plasmids		This study
m4104	CA rich	CACCACACCCACACACCACACCCACA	cDNA production		(Iglesias et al. 2011)
m4101	ACT1	GTAACATCGTTATGTCCGGTGGTAC	RT-qPCR internal control	97% (CT:13-27)	(Iglesias et al. 2011)
m4103	ACT1	CCAAGATAGAACCACCAATCCAGAC	cDNA production RT-qPCR internal control		(Iglesias et al. 2011)
m3275	BUD6	GACCGGGCACATTTAATCAG	RT-qPCR internal control	82% (CT: 18-31)	This study
m3276	BUD6	TCAGCCTTGTCAATAGCTTCG	RT-qPCR internal control		This study
m3556	TEL01L	CGGTGGGTGAGTGGTAGTAAGTAGA	TERRA RNA measurements	98% (CT:16-27)	(Balk et al. 2013)
m3557	TEL01L	ACCCTGTCCCATTCAACCATAC	TERRA RNA measurements		(Balk et al. 2013)
m4089	TEL10R	CGGTTATGGTGGACGGTGGATG	TERRA RNA measurements	92% (CT:14-28)	(Iglesias et al. 2011)
m4090	TEL10R	CCTAACCCTATTCTAATCCAACCCTGATAA	TERRA RNA measurements Southern Blot probe		(Iglesias et al. 2011)
m4093	TEL13R	ACGGTTATGGTGCACGATGGG	TERRA RNA measurements	106% (CT:14-27)	(Iglesias et al. 2011)
m4094	TEL13R	TTACCCTCCATTACGCTACCTCC	TERRA RNA measurements		(Iglesias et al. 2011)
m2335	TEL15L	TATCCTACTCCACTGCCACTTACCCTG	TERRA RNA measurements Southern Blot probe	92% (CT:16-27)	This study
m2336	TEL15L	TGTTAGCGTTTCAATATGGTGGGTAGA	TERRA RNA measurements		This study
m4105	TEL10R	TGACTTAACCTTGGCAGCTTC	Southern Blot probe		This study

m4113	TEL13R	TGGGCTTTATGGGTAAATGG	Southern Blot probe		This study
m4116	TEL13R	TACCCTGATTTAGCATGTCTCTTA	Southern Blot probe		This study
m4118	TEL15L	AGTGGAAACGTGATAAACTGC	Southern Blot probe		This study
m4269	YOL166W-A (TEL15L)	GCTTGCCTCAGCGGTCTAT	TERRA and Silencing measurements	94% (CT:13-24)	This study
m4270	YOL166W-A (TEL15L)	TGGGCCGCCAAATGAGATA	TERRA and Silencing measurements		This study
m4273	YAL068W-A (TEL01L)**	TACCCATAACGCCCATCATT	TERRA and Silencing measurements	98% (CT:17-28)	This study
m4274	YAL068W-A (TEL01L)**	TGGTGCAAAAGTGGTATAACG	TERRA and Silencing measurements		This study
m4275	7S RNA	GGCAGGAGGCGTGAGGAATC	RT-qPCR internal control	108.99% (CT:12-28)	(Iglesias et al. 2011)
m4276	7S RNA	CCTAACAGCGGTGAAGGTGGAG	RT-qPCR internal control		(Iglesias et al. 2011)
m2245	TEL06R	CGTATGCTAAAGTATATATTACTTCACTCCATT	ChIP	91% (CT:20-31)	(Holstein et al. 2014)
m2246	TEL06R	TCCGAACTCAGTTACTATTGATGGAA	ChIP		(Holstein et al. 2014)
m4623	*CEN3*	GATCAGCGCCAAACAATATGGAAAATCC	ChIP	82% (CT:15-31)	(Lefrancois et al. 2013)
m4624	*CEN3*	AACTTCCACCAGTAAACGTTTCATATATCC	ChIP		(Lefrancois et al. 2013)
m4099	Y'3***	GGCTTGGAGGAGACGTACATG	ChIP	89% (CT:10-24)	(Iglesias et al. 2011)
m4100	Y'3***	CCACACACTCTCTCACATCTACCTC	ChIP		(Iglesias et al. 2011)

*Serial dilutions of genomic DNA (gDNA) were measured by qPCR using all qPCR primer pairs. CT values were plotted against the log of the gDNA concentration. The slope of the regression line that best fitted data was used to calculate primer efficiencies (E). E = (10^(–1/slope) –1) x 100. The CT intervals, in brackets, correspond to the range used to calculate primer efficiencies.
**Could also recognise TEL03L (with 1 mismatch for both forward and reverse primers). Melting curves show only one peak.
***Conserved across 3 different Y' telomeres.

Table S3 – List of plasmids used.

pDL	Alias	Ref.
751	pSD120	(Diede and Gottschling 1999)
1713	pRS316	(Sikorski and Hieter 1989)

Supplementary references

Addinall SG, Holstein EM, Lawless C, Yu M, Chapman K, Banks AP, Ngo HP, Maringele L, Taschuk M, Young A et al. 2011. Quantitative fitness analysis shows that NMD proteins and many other protein complexes suppress or enhance distinct telomere cap defects. *Plos Genet* **7**: e1001362.

Balk B, Maicher A, Dees M, Klermund J, Luke-Glaser S, Bender K, Luke B. 2013. Telomeric RNA-DNA hybrids affect telomere-length dynamics and senescence. *Nat Struct Mol Biol* **20**: 1199-+.

Diede SJ, Gottschling DE. 1999. Telomerase-mediated telomere addition in vivo requires DNA primase and DNA polymerases alpha and delta. *Cell* **99**: 723-733.

Haruki H, Nishikawa J, Laemmli UK. 2008. The anchor-away technique: rapid, conditional establishment of yeast mutant phenotypes. *Molecular cell* **31**: 925-932.

Holstein EM, Clark KR, Lydall D. 2014. Interplay between nonsense-mediated mRNA decay and DNA damage response pathways reveals that Stn1 and Ten1 are the key CST telomere-cap components. *Cell reports* **7**: 1259-1269.

Iglesias N, Redon S, Pfeiffer V, Dees M, Lingner J, Luke B. 2011. Subtelomeric repetitive elements determine TERRA regulation by Rap1/Rif and Rap1/Sir complexes in yeast. *EMBO reports* **12**: 587-593.

Lefrancois P, Auerbach RK, Yellman CM, Roeder GS, Snyder M. 2013. Centromere-like regions in the budding yeast genome. *Plos Genet* **9**: e1003209.

Maringele L, Lydall D. 2004. EXO1 plays a role in generating type I and type II survivors in budding yeast. *Genetics* **166**: 1641-1649.

Morin I, Ngo HP, Greenall A, Zubko MK, Morrice N, Lydall D. 2008. Checkpoint-dependent phosphorylation of Exo1 modulates the DNA damage response. *The EMBO journal* **27**: 2400-2410.

Sikorski RS, Hieter P. 1989. A system of shuttle vectors and yeast host strains designed for efficient manipulation of DNA in Saccharomyces cerevisiae. *Genetics* **122**: 19-27.

Supplementary Figure S1

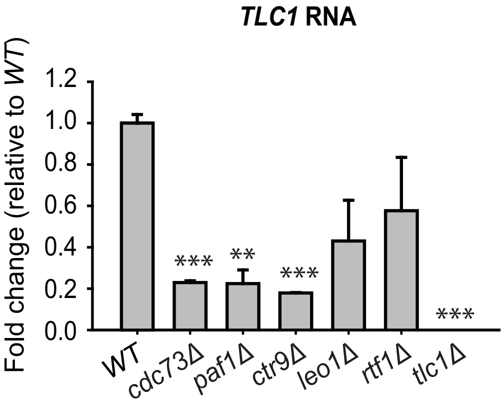

Supplementary Figure S2

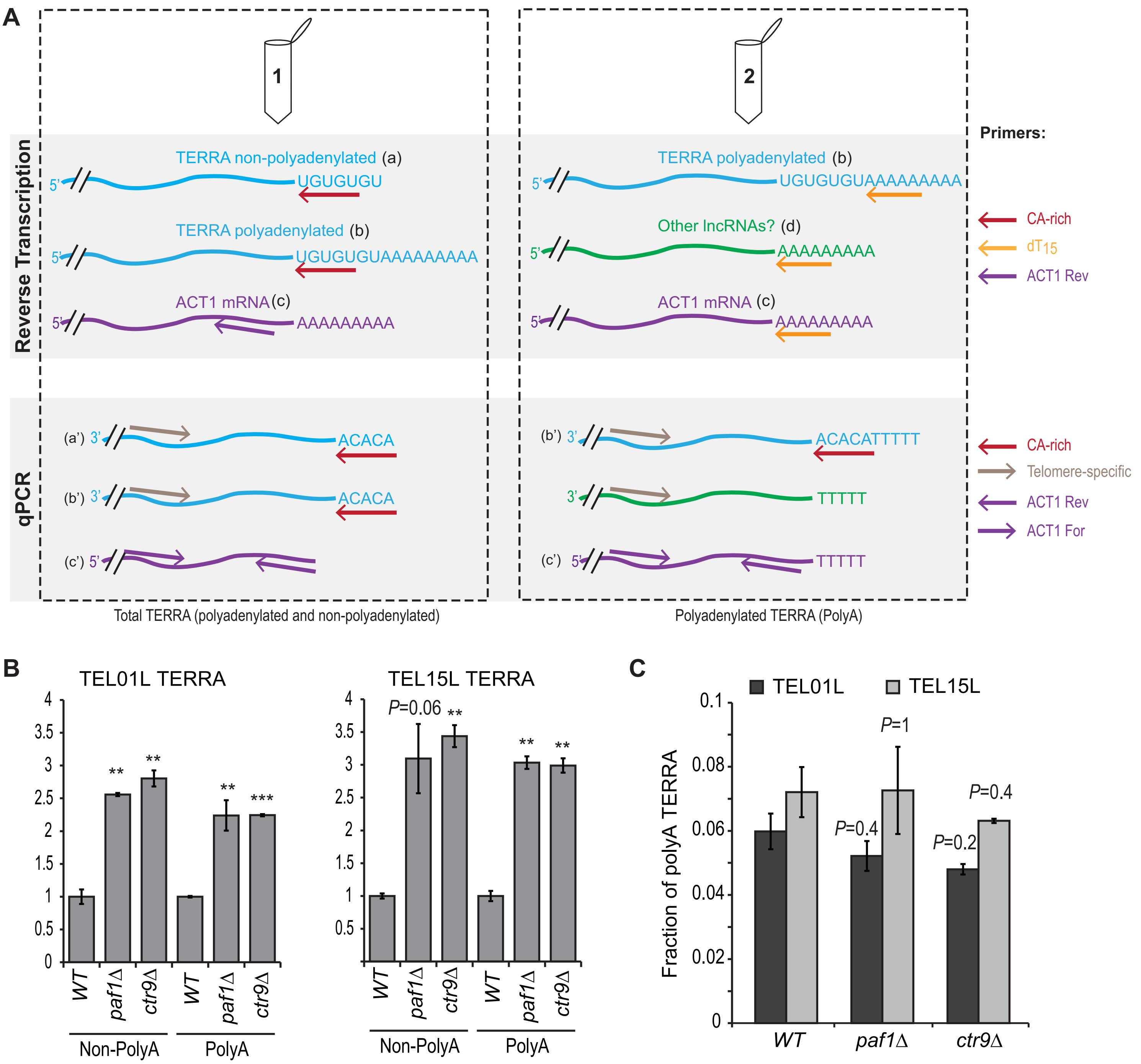

D

		Average CT			Average CT		
		ACT1	***1L***	**2^(-(1L-ACT1))**	***ACT1***	***15L***	**2^(-(15L-ACT1))**
Total	*WT*	11.55	22.01	7.10E-04	11.00	21.06	9.35E-04
	WT	11.67	21.83	8.78E-04	11.02	20.99	9.95E-04
	paf1Δ	11.77	20.70	2.04E-03	11.07	19.70	2.52E-03
	paf1Δ	11.92	20.90	1.99E-03	11.30	19.48	3.45E-03
	ctr9Δ	11.94	20.83	2.11E-03	11.27	19.59	3.13E-03
	ctr9Δ	12.04	20.81	2.29E-03	11.35	19.53	3.44E-03
PolyA	*WT*	11.96	26.36	4.64E-05	11.37	25.08	7.47E-05
	WT	12.10	26.45	4.76E-05	11.50	25.44	6.39E-05
	paf1Δ	12.26	25.33	1.16E-04	11.68	23.85	2.17E-04
	paf1Δ	12.29	25.66	9.43E-05	11.83	24.09	2.04E-04
	ctr9Δ	12.18	25.40	1.05E-04	11.65	23.94	2.00E-04
	ctr9Δ	12.47	25.67	1.06E-04	11.86	24.04	2.15E-04

Supplementary Figure S3

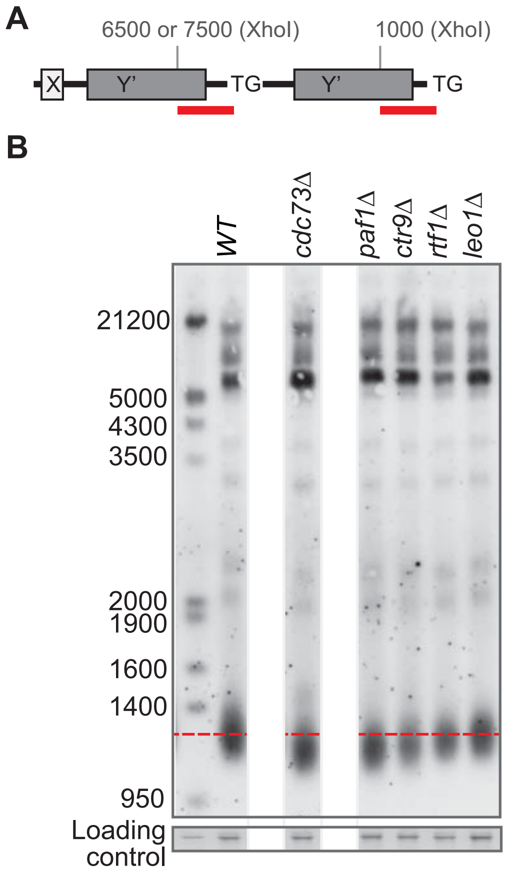

Supplementary Figure S4

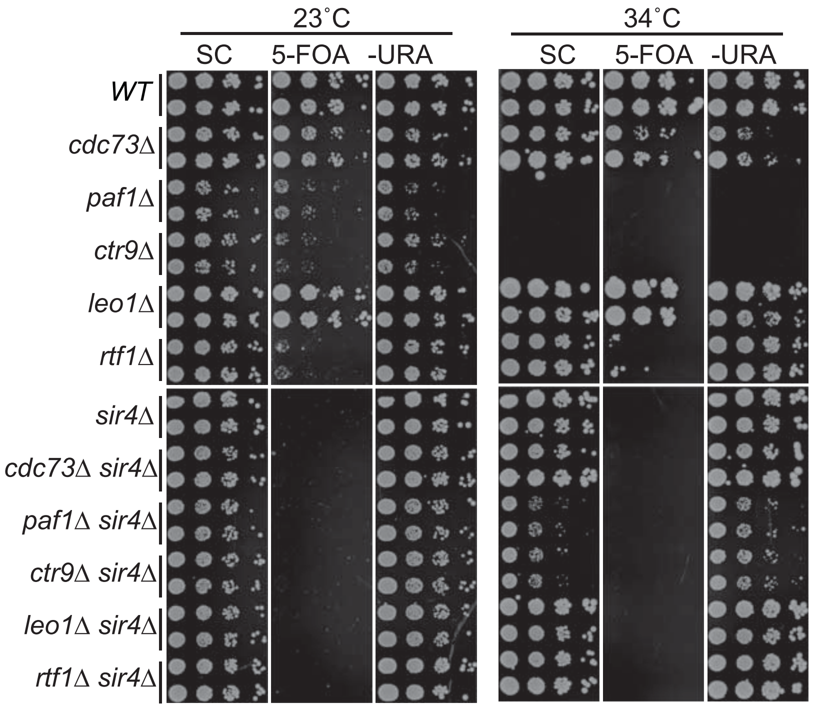

Supplementary Figure S5

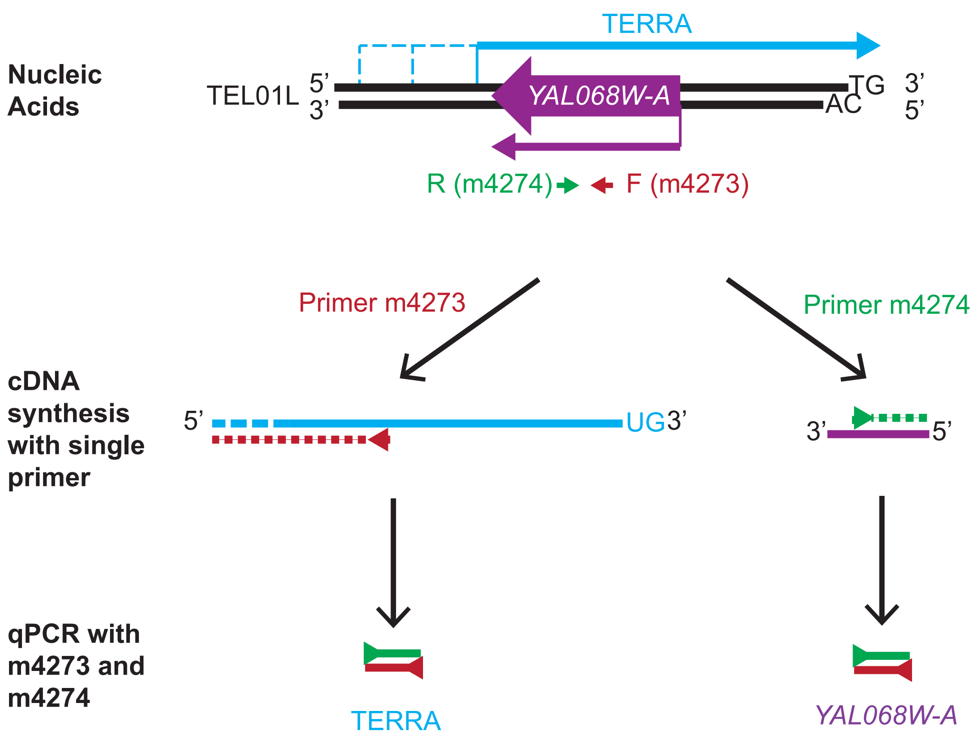

Supplementary Figure S6

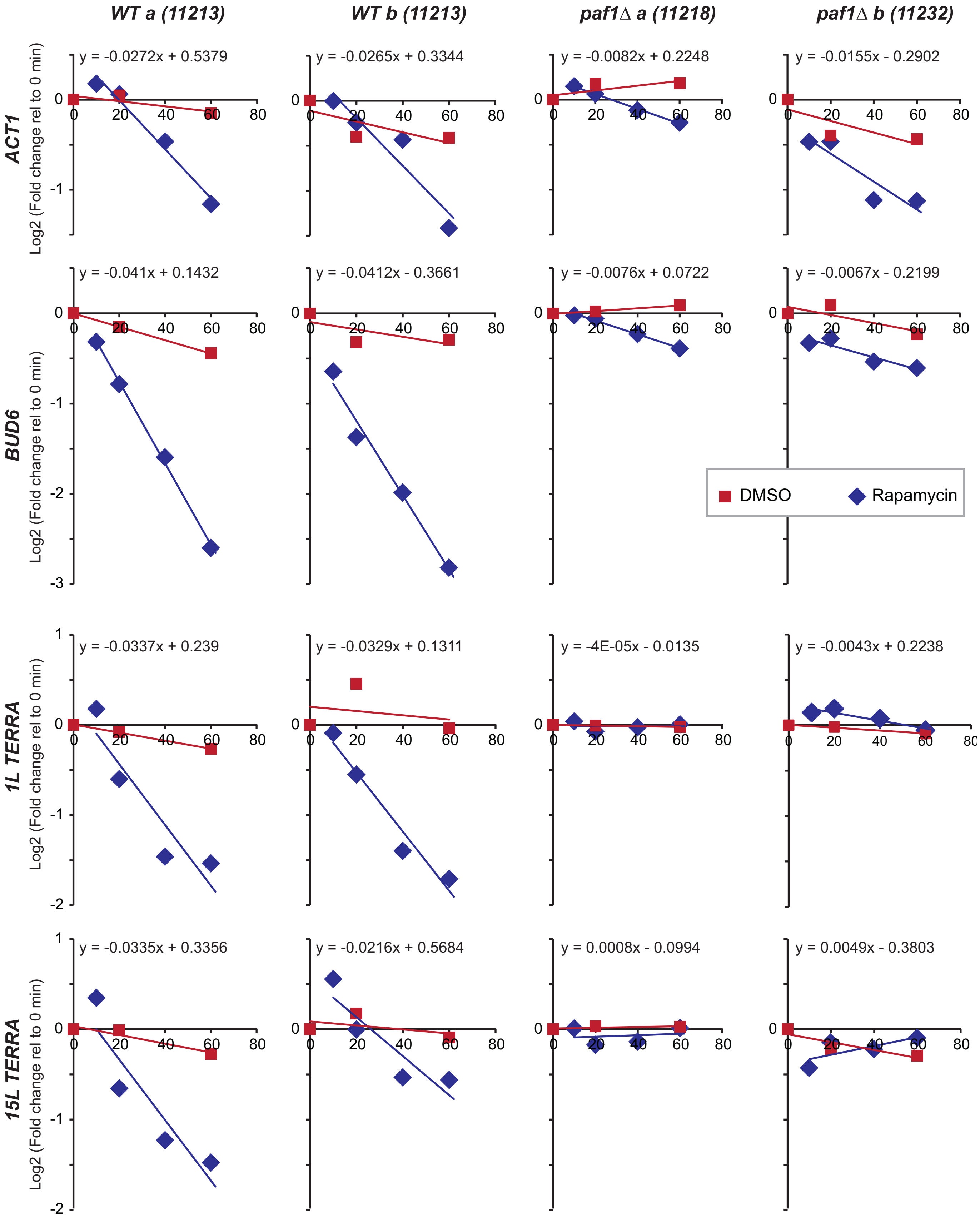

Supplementary Figure S7

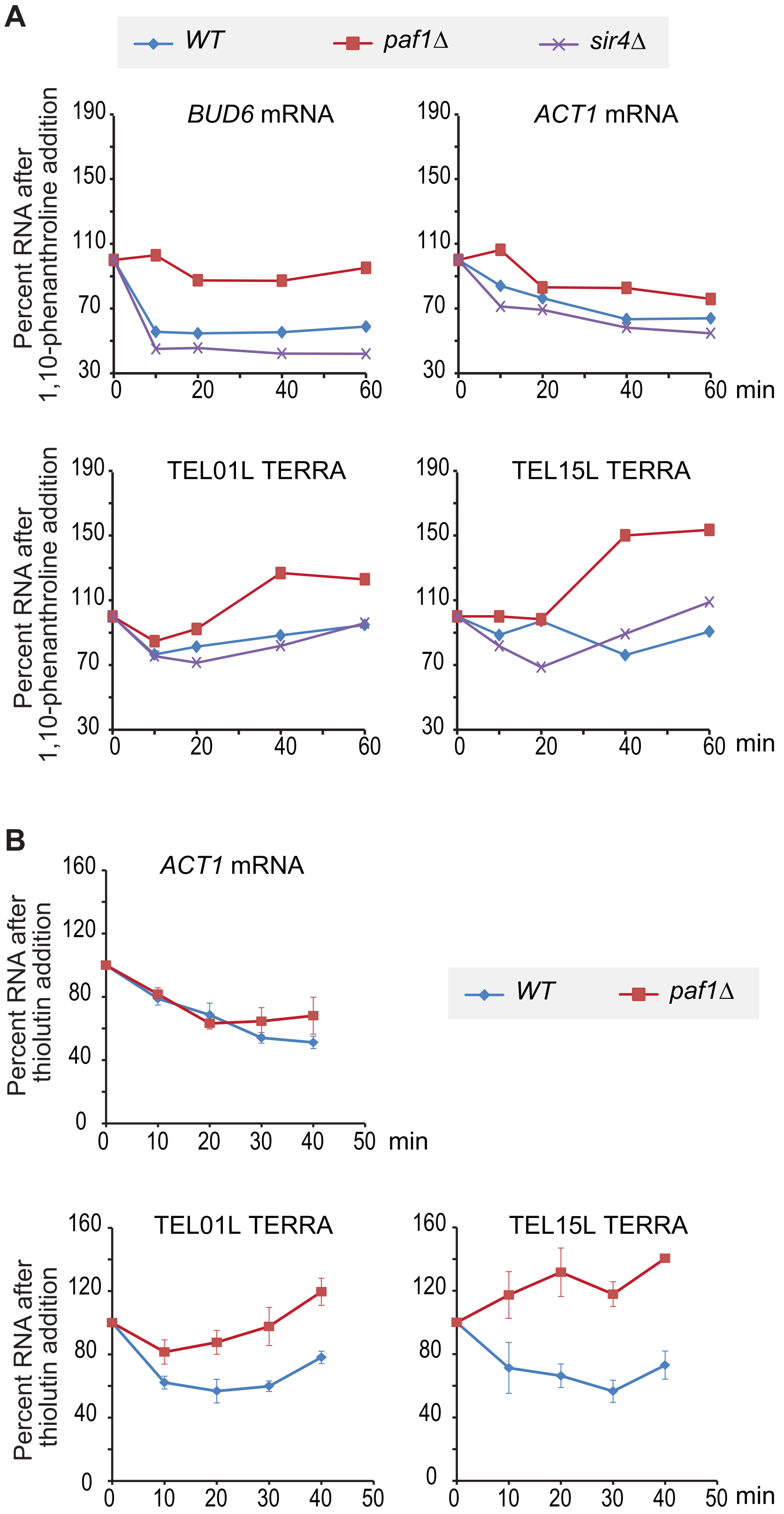

Supplementary Figure S8

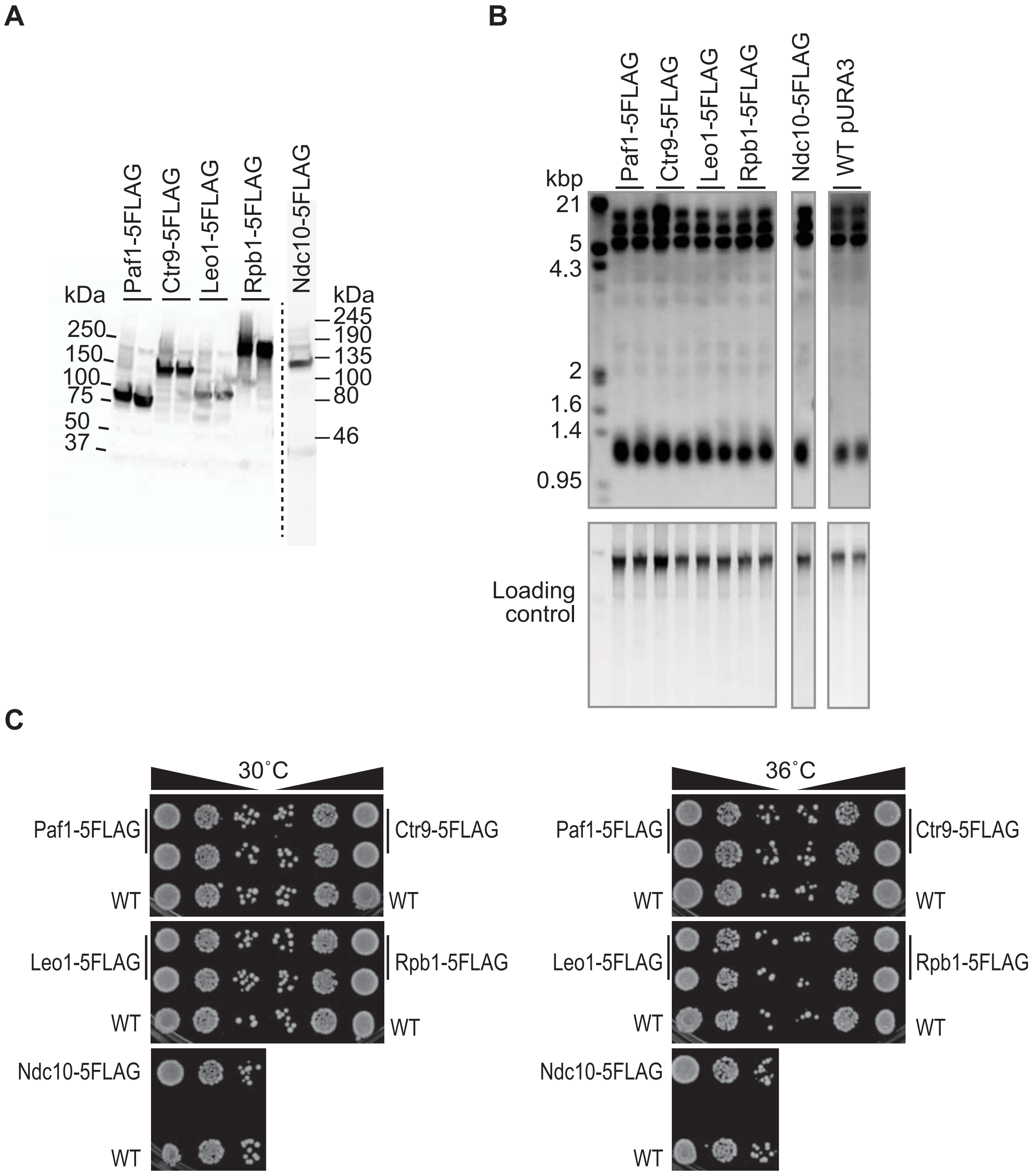

Supplement: Supplementary Data [file gkx1131_supp.pdf]
